# Osmotic stress is accompanied by protein glycation in *Arabidopsis thaliana*

**DOI:** 10.1093/jxb/erw395

**Published:** 2016-11-10

**Authors:** Gagan Paudel, Tatiana Bilova, Rico Schmidt, Uta Greifenhagen, Robert Berger, Elena Tarakhovskaya, Stefanie Stöckhardt, Gerd Ulrich Balcke, Klaus Humbeck, Wolfgang Brandt, Andrea Sinz, Thomas Vogt, Claudia Birkemeyer, Ludger Wessjohann, Andrej Frolov

**Affiliations:** ^1^Department of Bioorganic Chemistry, Leibniz Institute of Plant Biochemistry, Halle (Saale), Germany; ^2^Faculty of Chemistry and Mineralogy, Universität Leipzig, Leipzig, Germany; ^3^Department of Plant Physiology and Biochemistry, St Petersburg State University, St Petersburg, Russia; ^4^Department of Pharmaceutical Chemistry and Bioanalytics, Institute of Pharmacy, Martin-Luther Universität Halle-Wittenberg, Halle (Saale), Germany; ^5^Department of Plant Physiology, Martin-Luther Universität Halle-Wittenberg, Halle (Saale), Germany; ^6^Department of Metabolic and Cell Biology, Leibniz Institute of Plant Biochemistry, Halle (Saale), Germany

**Keywords:** Advanced glycation end-products (AGEs), *Arabidopsis thaliana*, crop quality, drought stress, food quality, glycation, label-free quantification, plant proteomics, two-dimensional chromatography.

## Abstract

Among the environmental alterations accompanying oncoming climate changes, drought is the most important factor influencing crop plant productivity. In plants, water deficit ultimately results in the development of oxidative stress and accumulation of osmolytes (e.g. amino acids and carbohydrates) in all tissues. Up-regulation of sugar biosynthesis in parallel to the increasing overproduction of reactive oxygen species (ROS) might enhance protein glycation, i.e. interaction of carbonyl compounds, reducing sugars and α-dicarbonyls with lysyl and arginyl side-chains yielding early (Amadori and Heyns compounds) and advanced glycation end-products (AGEs). Although the constitutive plant protein glycation patterns were characterized recently, the effects of environmental stress on AGE formation are unknown so far. To fill this gap, we present here a comprehensive in-depth study of the changes in *Arabidopsis thaliana* advanced glycated proteome related to osmotic stress. A 3 d application of osmotic stress revealed 31 stress-specifically and 12 differentially AGE-modified proteins, representing altogether 56 advanced glycation sites. Based on proteomic and metabolomic results, in combination with biochemical, enzymatic and gene expression analysis, we propose monosaccharide autoxidation as the main stress-related glycation mechanism, and glyoxal as the major glycation agent in plants subjected to drought.

## Highlight

Osmotic stress enhances the rate of protein glycation and monosaccharide autoxidation is the main pathway.

## Introduction

Environmental stress is one of the major factors reducing crop plant productivity all over the world, and drought represents its most important manifestation (Cruz de Carvalho, 2008). The oncoming climate changes are making drought, usually defined as a period of lower than normal precipitation that limits a plant’s productivity in a natural or agricultural system (Brunner *et al.*, 2015), an important economic factor directly influencing the yield and quality of crops. Additionally, several other environmental stress factors like high salinity, reduced or increased temperatures, as well as their combinations, result in a reduced water availability and cause the appearance of drought symptoms (Verslues *et al.*, 2006). On the physiological level, water deficiency triggers reduction of stomatal aperture, decrease of CO_2_ assimilation rate and growth inhibition (Chaves *et al.*, 2009). This leads to an imbalance between the generation of reduction equivalents by the light reactions and their consumption in the Calvin cycle. In turn, this increases the rates of single electron reduction of molecular oxygen by the redox proteins of the chloroplasts and mitochondrial electron transport chains and enhances production of reactive oxygen species (ROS): first, singlet oxygen (^1^O_2_) and superoxide anion radical (O_2_^•−^), and further, hydrogen peroxide (H_2_O_2_), nitric oxide (NO) and the hydroxyl radical (^•^OH) (Kar, 2011). When production of these ROS overwhelms their detoxification, oxidative stress develops (Gill and Tuteja, 2010), and the rates of lipid peroxidation (Mirzaee *et al.*, 2013), monosaccharide autoxidation (Wolff and Dean, 1987), and oxidative degradation of proteins (Berlett and Stadtman, 1997) increase. The generated products of lipid and sugar oxidative degradation, hydroxycarbonyls and α-oxocarbonyls, are potent protein modification agents and can trigger essential changes in the structure and function of polypeptides (Lennicke *et al.*, 2016).

**Fig. 1.**
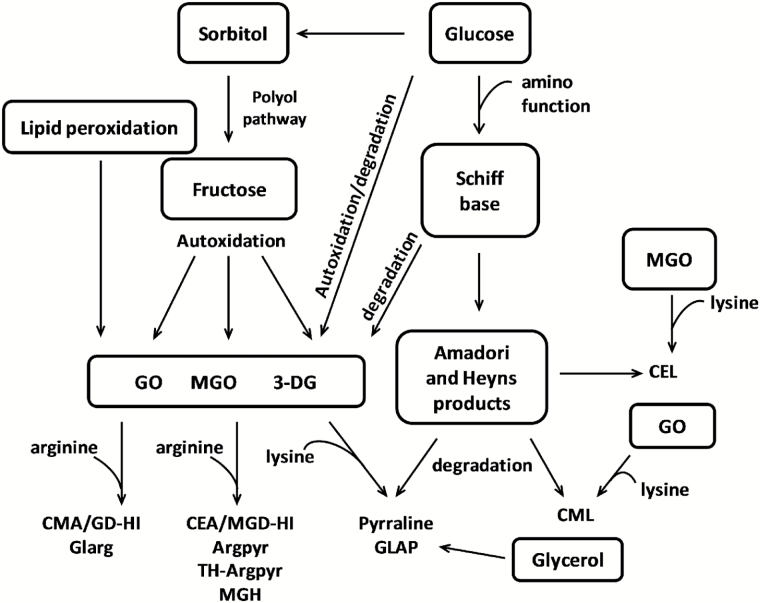
Pathways of AGE formation.

As the plant stress response is accompanied by rapid accumulation of mono- and oligosaccharides (Arbona *et al.*, 2013), as well as increases in the concentration of some amino acids and peptides, glycation might contribute significantly to stress-related protein damage (Bechtold *et al.*, 2009). Indeed, reducing sugars (both aldoses and ketoses) interact with protein amino groups, yielding the corresponding keto- and aldamines (i.e. Amadori and Heyns compounds; Hodge, 1955; Heyns and Noack, 1962). These early glycation products, as well as free sugars, readily autoxidize to yield highly reactive α-dicarbonyl compounds, presumably glyoxal (GO), methylglyoxal (MGO) and 3-deoxyglucosone (3-DG), potent intermediates of advanced glycation (Fig. 1; Wolff and Dean, 1988). Their interaction with lysyl amino and arginyl guanidinium groups results in formation of so-called advanced glycation end-products (AGEs) – protein Maillard compounds also found to accumulate during thermal processing of foods (Greifenhagen *et al.*, 2015). Upon their absorption in the human intestine, AGEs interact with endothelial and macrophage pattern recognition receptors for AGEs (e.g. RAGEs) and trigger nuclear factor-κB (NF-κB)-mediated expression of pro-inflammatory molecules (Goldin *et al.*, 2006), thus contributing to the local and systemic subclinical inflammation underlying atherosclerosis and accompanying pathologies (Nettleton *et al.*, 2007).

Over the last decades, multiple biologically relevant AGEs were reported. Among them, *N* ^ε^-(carboxymethyl)lysine (CML; van de Kerkhof *et al.*, 2004), *N* ^ε^-(carboxyethyl)lysine (CEL; Zhu *et al.*, 2012), ɛ-(2′-formyl-5′-hydroxymethyl-pyrrolyl)-L-norleucine (pyrraline; Foerster and Henle, 2003), and glyceraldehyde-derived pyridinium (GLAP; Usui *et al.*, 2007) are the best-characterized lysine-derived products. For arginine residues, Schwarzenbolz and coworkers described 1-(4-amino-4-carboxybutyl)-2-imino-5-oxo-imidazolidine (Glarg) formed in the reaction with glyoxal (Schwarzenbolz *et al.*, 1997) and yielding *N* ^δ^-carboxylethylarginine (CMA) under physiological conditions (Glomb and Lang, 2001). Methylglyoxal forms methylglyoxal-derived hydroimidazolones (MG-Hs) with *N* ^δ^-(5-methyl-4-oxo-5-hydroimidazolinone-2-yl)-L-ornithine (MG-H1) as the major representative (Henle *et al.*, 1994). Hydrolysis of another MG-H, namely 2-amino-5-(2-amino-4-hydro-4-methyl-5-imidazolon-1-yl)pentanoic acid (MG-H3) yields carboxyethyl-L-arginine (CEA) (Gruber and Hofmann, 2005). Sequential modification of an arginine with two methylglyoxal molecules results in *N* ^δ^-(5-hydroxy-4,6-dimethylpyrimidine-2-yl)-L-ornithine (argpyrimidine, Argpyr; Shipanova *et al.*, 1997) and *N* ^δ^-(4-carboxy-4,6-dimethyl-5,6-dihydroxy-1,4,5,6-tetrahydropyrimidine-2-yl)-L-ornithine (tetrahydropyrimidine, TH-Pyr; Oya *et al.*, 1999).

A few years ago, Bechtold *et al.* (2009) reported an increase in the total content of different individual AGE classes (detected as glycated amino acids after exhaustive hydrolysis) upon the application of experimental environmental stress to Arabidopsis plants. Most recently, Bilova *et al.* (2016) characterized hundreds of constitutively glycated proteins in Arabidopsis and *Brassica napus*. However, the effects of environmental changes on the plant glycated proteome were not considered so far. To fill this gap, we describe here the effects of short-term osmotic stress on the advanced glycation patterns in the Arabidopsis proteome and characterize, to the best of our knowledge, for the first time the group of stress-specifically AGE-modified proteins along with the pathways of advanced glycation.

## Materials and methods

### Reagents

Unless stated otherwise, materials were obtained from the following manufacturers: AAT Bioquest^®^, Inc. (Sunnyvale, CA, USA): Amplite Fluorimetric Glutathione GSH/GSSG Ratio Assay Kit; AppliChem GmbH (Darmstadt, Germany): Tris (ultrapure); Biosolve (Valkenswaard, The Netherlands): acetonitrile (MS grade); Biomol GmbH, Hamburg, Germany: goat anti-2,4-dinitrophenylhydrazine (DNP) antibody, donkey anti-goat IgG H&L DyLight® 488 conjugated; Biozym Scientific GmbH (Hessisch Oldendorf, Germany): 200 µl polypropylene tubes, microporous low-fluorescence polyvinylidene fluoride (PVDF) membrane, AdvanBlock^TM^-PF protein-free blocking and AdvanWash^TM^ Immonoblot washing solutions; Carl Roth GmbH & Co KG (Karlsruhe, Germany): agar-agar Kobe I, polyethylene glycol (PEG) 8000 (p.a.), 2-(*N*-morpholino)ethansulfonic acid (MES) (p.a.), sodium dodecylsulfate (≥99.5%), tris-(2-carboxyethyl)phosphine hydrochloride (TCEP, ≥98%), D-glucose monohydrate (≥99.5%), D-(–)-fructose (≥99.5%), D-(+)-sucrose (≥99.5%), and glycerin (≥99.5%); Conlac GmbH (Leipzig, Germany): hexane (puriss); Dichrom GmbH (Marl, Germany): Progenta™ anionic acid labile surfactant II; Macherey-Nagel GmbH & Co KG (Düren, Germany): *N*-methyl-*N*-(trimethylsilyl) trifluoroacetamide (MSTFA, MS grade), Chromabond^®^ XR-XC sorbent, Chromabond^®^ Multi 96 microtiter plate with 20 µm filters; Merck KGaA (Darmstadt, Germany): L-(+)-arabinose (≥99%), D-(+)-maltose monohydrate (≥98%), D-(+)-mannose (≥98%); Perbio Science Deutschland GmbH (Bonn, Germany): PageRuler™ Plus Prestained Protein Ladder, 10 to 250 kDa; Santa Cruz Biotechnology Inc. (Heidelberg, Germany): D_6_-abscisic acid (pure); SERVA Electrophoresis GmbH (Heidelberg, Germany): acrylamide/bis-acrylamide solution (37.5/1, 30% (w/v), 2.6% C), ammonium persulfate (p.a.), *N*,*N*,*N*′,*N*′-tetramethylethane-1,2-diamine (research grade), Coomassie Brilliant Blue G 250 (pure), glycine (p.a.), sucrose (>98%) and NB sequencing grade modified trypsin from porcine pancreas; VWR International GmbH (Dresden, Germany): D-(+)-galactose, D-(–)-ribose, D-(–)-mannitol, *myo*-inositol, and acetonitrile (≥99.9%, HPLC grade); Ferak Berlin (Berlin, Germany): trichloroacetic acid (TCA). All other chemicals were purchased from Sigma-Aldrich Chemie GmbH (Taufkirchen, Germany). Water was purified in house (resistance>18 mΩ cm^–1^; total organic content<1 ppb) on a PureLab Ultra Analytic System (ELGA Lab Water, Celle, Germany).

### Plant experiments

Germination and plant growth were accomplished under sterile conditions in culture boxes (Greiner Bio-One GmbH, Frickenhausen, Germany), equipped with 96-well polypropylene trays (SARSTEDT AG & Co, Nürnbrecht, Germany) and filled with 200 ml with half-strength Murashige and Skoog medium supplemented with 0.5% sucrose, pH 5.7. Two-hundred-microliter polypropylene tubes were filled with 0.8% agar in half-strength Murashige and Skoog medium, cut and placed in the wells of the 96-well trays. The seeds of wild type *Arabidopsis thaliana* (Columbia) were planted under sterile conditions in agar-filled tubes. The plants were grown for 2 weeks in a phytotron MLR-351H (SANYO Electric Co., Ltd, Moriguchi, Japan) at 23 ºC (day) and 18 ºC (night) under short day with an 8 h light (112.5 ± 2.5 µmol photons m^−2^ s^−1^)–16 h dark cycle and 60% humidity before transfer to 50 ml brown glass bottles filled with half-strength Murashige and Skoog medium (pH 5.7). After the 4-week growth under the same conditions, the plants were transferred to agar (200 ml) supplemented with the same medium in 6 mmol l^–1^ MES buffer (pH 5.7) and filled in polypropylene pots (Combiness Europe, Nazareth, Belgium). Prior to the plant transfer, the agar was overlaid for 3 d with 300 ml of MES-buffered Murashige and Skoog medium with and without addition of 172.27 g l^–1^ PEG8000 to provide drought stress and control conditions, respectively (Money, 1989). (A detailed description of model establishment and optimization of the stressor dosage are given in Frolov *et al.*, 2016) The plants were harvested 3 d after the transfer to agar. The leaves were firstly ground under liquid nitrogen by mortar and pestle and later in a Mixer Mill MM 400 ball mill with Ø 3 mm stainless steel balls (Retsch, Haan, Germany) at a vibration frequency of 30 Hz for 2 × 1 min.

### Determination of water potential

Leaf water potential (ψ_w_) was determined by the gravimetric method of Rayle *et al.* (1982) with modifications. In detail, individual leaves were weighed and immediately submerged for 2 h at room temperature (RT) in 4.5 ml of sucrose solutions (0.1–1 mol l^–1^) in Petri dishes (2 cm diameter) covered with glass slides. Afterwards, the leaves were carefully blotted, weighed, and the weight loss or gain was documented. The ψ_w_ values were calculated by the Van’t Hoff equation (ψ_w_=–*iMRT*), where *M* denotes the sugar molar concentration yielding no changes in leaf weight. The ψ_w_ values of the agar medium (collected into 1.5 ml polypropylene tubes from the surface and bottom of each pot) were determined by vapor pressure osmometer (Vapro 5520, Wescor Inc., Logan, UT, USA).

### Physiological and biochemical assays

The stomatal conductance, PS II efficiency (variable fluorescence (*F*_v_)/maximal fluorescence (*F*_m_)), and relative chlorophyll content were determined by means of a diffusion porometer (Delta-T Devices Ltd, Cottbus, Germany), photosynthesis yield analyzer (Walz GmbH, Effeltrich, Germany), and chlorophyll meter (Konica Minolta, Langenhagen, Germany), respectively, according to established protocols (Morison, 1998; Silva *et al.*, 2007) using the three youngest leaves (11th–13th) of each plant (*n*=9). The fresh and dry leaf weights were determined for all plant leaves before and after a 3 d incubation at 80 °C, respectively. The leaf relative water content (LRWC) was calculated as follows: LRWC (%)=(fresh weight–dry weight)/fresh weight×100.

Lipid hydroperoxides were quantified by oxidation of a Fe(II)-xylenol orange complex as described by Griffiths *et al.* (2000) with modifications (Supplementary Protocol S1 at *JXB* online). Determination of H_2_O_2_ contents relied on the modified method of Gay *et al.* (1999) (Supplementary Protocol S2). Malondialdehyde (MDA) contents were determined as described by Velikova *et al.* (2000) using approximately 25 mg of plant material (Supplementary Protocol S3). Ascorbic and dehydroascorbic acids were quantified in leaf tissue as described by Huang *et al.* (2005) with minor modifications (Supplementary Protocol S4). Reduced and oxidized glutathione (GSH and GSSG, respectively) and glyoxalase II activity were quantified as described by Bilova *et al.* (2016). Analysis of leaf abscisic acid (ABA) contents was based on the method of Balcke *et al.* (2012). Gene expression analysis was performed by RT-qPCR using commercially available kits and results were calculated according to Schmittgen and Livak (2008) (Supplementary Protocol S5). Details of primer sequence of genes are summarized in Supplementary Table S1.

### Protein isolation

The total protein content was isolated by phenol extraction as described by Isaacson *et al.* (2006). In detail, 650 µl of ice-cold phenol extraction buffer (0.7 mol l^–1^ sucrose, 0.1 mol l^–1^ KCl, 5 mmol l^–1^ EDTA, 2% (v/v) mercaptoethanol and 1 mmol l^–1^ protease inhibitor mixture in 0.5 mol l^–1^ Tris–HCl buffer, pH 7.5) was added to approximately 250 mg of plant material and vortexed for 30 s. Directly afterwards, 650 µl of phenol saturated with 0.5 mol l^–1^ Tris–HCl buffer (pH 7.5) was added and the suspension was shaken for 30 min at 900 r.p.m. (4 °C) and centrifuged at 5000 *g* for 30 min at 4 °C. The upper phenol phase was collected and supplemented with an equal volume of phenol extraction buffer. Water-soluble substances were re-extracted twice as described above. Finally, five volumes (1 ml) of ice-cold 0.1 mol l^–1^ ammonium acetate in methanol was added to the collected phenol phase (approximately 200 µl) and the samples were left at –20 °C overnight. Next morning, the samples were centrifuged for 30 min at 5000 *g* (4 °C) and supernatants were discarded. The pellets were washed two times with ice-cold methanol and once with acetone (resuspended by vortexing for 3 min followed by centrifugation at 5000 *g* for 10 min) and dried by air flow under the hood.

### Tryptic digestion

Proteins were reconstituted in 200 µl of 100 mmol l^–1^ Tris–HCl (pH 7.5) containing 8 mol l^–1^ urea, 2 mol l^–1^ thiourea and 0.2% Progenta acidic anionic surfactant II. Protein concentrations were determined by the Bradford assay in 96-well plate format as described elsewhere (Frolov *et al.*, 2014*a*) (Supplementary Protocol S6). Protein (200 µg) was complemented with TCEP (50 mmol l^–1^, 10 µl), diluted with aqueous ammonium hydrogen carbonate solution (50 mmol l^–1^) to obtain a final volume of 100 µl, and incubated at 37 °C for 30 min. The samples were cooled to RT, alkylated with iodoacetamide (0.1 mol l^–1^, 11 µl) in the darkness for 60 min at 4 °C, and diluted eight-fold with 50 mmol l^–1^ ammonium bicarbonate solution. Trypsin (0.4 µg µl^–1^ in 50 mmol l^–1^ ammonium hydrogen carbonate) was added sequentially two times (in 1:20 and 1:50 enzyme/substrate ratio) and incubations were performed at 37 °C for 5 and 12 h, respectively. Afterwards, 10% (v/v) aq. trifluoroacetic acid (UV grade) was added to give a final concentration of 1% (v/v), and the digests were incubated at 37 °C for 20 min to destroy the Progenta anionic surfactant. Afterwards, the completeness of the digestion was determined by SDS-PAGE analysis (Supplementary Protocol S7). The digests were loaded on Oasis HLB cartridges (10 mg, 30 µm; Waters GmbH, Eschborn, Germany) mounted on the Chromabond vacuum manifold (Macherey Nagel, Düren, Germany). After washing with 1 ml 0.1% (v/v) aqueous formic acid, retained tryptic peptides were subsequently eluted with 333 µl of 40%, 60% and 80% (v/v) aq. acetonitrile. The combined eluates were concentrated under vacuum for 30 min, freeze-dried overnight, and stored at –20 °C.

### Western blotting

Anti-dinitrophenylhydrazine (DNP) Western blot analysis was performed as described by Fedorova *et al.* (2010), with modifications. The proteins separated by SDS-PAGE (Supplementary Protocol S7) were transferred electrophoretically (100 V, 60 min) to a PVDF membrane pre-activated with ethanol and equilibrated in 39 mmol l^–1^ glycine/48 mmol l^–1^ Tris buffer (pH 10.4) for 1 h. The membrane-bound protein carbonyls were derivatized with 1 mg ml^–1^ 2,4-dinitrophenylhydrazine in 2 mol l^–1^ HCl (30 min), washed with methanol and blocked for 1 h with AdvanBlock^TM^-PF protein-free blocking solution. Afterwards the membrane was treated with primary antibodies (Bethyl A150-117A goat anti-DNP, 1:30000, 1 h), intensively washed (3 × 10 min, AdvanWash^TM^ Immonoblot Washing Solution), and treated with secondary antibodies (Bethyl A50-201D2 donkey anti-goat IgG H&L DyLight® 488, 1:20000, darkness, 1 h). The carbonylated proteins were visualized by the gel imager Chemi Doc MP (Bio-Rad Laboratories GmbH, München, Germany) at λ_Ex_=493 nm, λ_Em_=518 nm by using the Bio-Rad Image Lab 5.1 software.

### Gas chromatography–mass spectrometry

Profiling of primary metabolites and α-dicarbonyls relied on established methods (Milkovska-Stamenova *et al.*, 2015; Bilova *et al.*, 2016) using the parameters summarized in Supplementary Table S2. The trimethylsilyl-methoxime derivatives of the analytes were annotated by retention time indexes (determined with C10–C32 alkanes) and mass spectral similarity search against the NIST08 database and an in-house library. Quantification of carbohydrates was performed by integration of the corresponding extracted ion chromatograms (*m*/*z*±0.5 Da) for representative intense signals at specific retention times (*t*_R_).

### Hydrophilic interaction liquid chromatography

Hydrophilic interaction liquid chromatography (HILIC) was performed as described by Singer *et al.* (2010) with modifications. In detail, tryptic digests (100 µg) were reconstituted in 40 µl of 0.1% (v/v) aq. formic acid before 260 µl of acetonitrile was added in three portions (60, 100 and 100 µl) followed by 30 s of vortexing after each addition. Of the sample, 290 µl was separated by a Luna 3u HILIC (silica, 100 × 2 mm, particle size 3 µm, Phenomenex, Aschaffenburg, Germany) using an Äkta Purifier HPLC system, equipped with A-905 autosampler, P-903 gradient pump binary pump, UV-900 UV-Vis detector, and Frac-950 fraction collector (GE Healthcare, Solingen, Germany). The separations were performed by linear gradients of aq. ammonium acetate in acetonitrile using the conditions summarized in Supplementary Table S3. The collected fractions (5 × 1 ml) were dried under reduced pressure, reconstituted in 60% (v/v) acetonitrile in 0.1% (v/v) aq. formic acid and diluted with 0.1% (v/v) formic acid (Supplementary Table S4).

### Nano-liquid chromatography–mass spectrometry analysis

The plant tryptic digests (70 ng) or individually diluted HILIC fractions were loaded on a nanoACQUITY UPLC^™^ Symmetry trap column (C_18_-phase, ID 180 μm, length 2 cm, particle size 5 µm) and then separated on a nanoACQUITY UPLC BEH130 column (C_18_-phase, ID 0.1 mm, length 10 cm, particle size 1.7 µm) using a nanoACQUITY UPLC system controlled by MassLynx X.4.1 software (Waters GmbH). The method settings and separating conditions are summarized in Supplementary Table S5. The column effluents were transferred via a PicoTip on-line nano-electrospray ionization (ESI) emitter to an LTQ Orbitrap XL electron-transfer dissociation mass spectrometer equipped with a nano-ESI source and controlled by Xcalibur 2.0.7 software (Thermo Fisher Scientific, Bremen, Germany). Analysis relied on a full mass spectrometry (MS) scan in the Orbitrap, followed by collision-induced dissociation fragmentation in the linear ion trap in data-dependent acquisition mode for the six most intense signals with charge states ranging from 2+ to 5+ using the MS settings summarized in Supplementary Table S6. For protein quantification, the LTQ Orbitrap XL-MS equipped with a nano-ESI source (Proxeon) was coupled on-line to a Dionex 3000 HPLC (Thermo Fisher Scientific). Identification of peptide sequences and protein annotation relied on the Sequest search against an Arabidopsis protein database (*Arabidopsis thaliana* proteome reviewed, Uniprot, http://www.ebi.ac.uk/ refe-rence_proteomes) using modification-specific mass increments and appropriate result filtering (Supplementary Table S6). The search was performed in automatic mode using the Proteome Discoverer 1.4 Software (Thermo Fisher Scientific) with false discovery rate of 0.01 (reversed decoy database). Different advanced glycation and oxidation modifications (Supplementary Table S6) were used in different combinations as search templates. The sequences of stress-specific peptides were confirmed by manual interpretation using the QualBrowser application of the Xcalibur 2.0.7 software as described elsewhere (Greifenhagen *et al.*, 2014; Schmidt *et al.*, 2015). Label-free quantification of AGE-modified peptides relied on integration of the corresponding extracted ion chromatograms (XICs) (*m*/*z*±0.02) at specific retention times using LCquan software (Thermo Fisher Scientific). The proteome quantitation was carried out with Progenesis QI software (Nonlinear Dynamics, Newcastle Upon Tyne, UK) using automatic alignment and peak picking (medium sensitivity) of individual HILIC fractions and subsequent combination of separate pre-fractions. Identification was performed with the MASCOT search engine using the Arabidopsis database (as mentioned above). The signals were normalized, using the total peak areas of one chromatographic analysis. Annotation of protein functions was performed by means of MapMan software (Max-Planck Institute of Molecular Plant Physiology, Potsdam-Golm, Germany, http://mapman.gabipd.org).

## Results

### Stress establishment and characterization

**Fig. 2.**
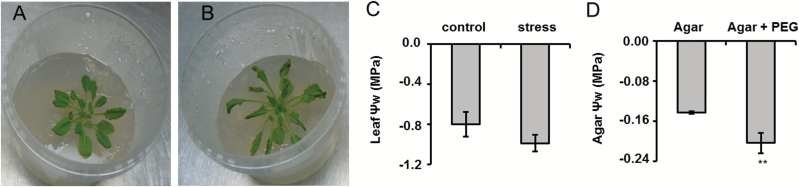
Arabidopsis plants grown for 3 d on a 0.8% agar medium infused with half-strength Murashige and Skoog medium in 6 mmol l^–1^ MES buffer (pH 5.7) in the absence (A) and presence (B) of 172.27 g l^–1^ PEG 8000 (overlay ψ _w_=–0.4 MPa), and ψ _w_ of plants (C) and corresponding agar medium (D) determined after plant harvesting. ** denotes statistical significance at the confidence levels *P*<0.01.

After 2 weeks’ growth on agar medium and 4 weeks’ growth in aq. culture, the plants developed a rosette of approximately 6–8 cm diameter and 0.5–1.0 g weight. On the third day after the plant transfer to agar saturated with Murashige and Skoog medium, no signs of osmotic stress could be seen. In contrast, clear loss of turgor was observed with the plants transferred to pots infused with PEG (Fig. 2A, B), although the water potential (ψ_w_) did not change in comparison with the controls (Fig. 2C). However, a 50% decrease of agar ψ_w_ upon saturation with PEG was registered (Fig. 2D, *t*-test, *P*=6.5 × 10^–3^). This reduction was equal in the whole agar volume (ψ_w_ relative standard deviation did not exceed 10%).

**Fig. 3.**
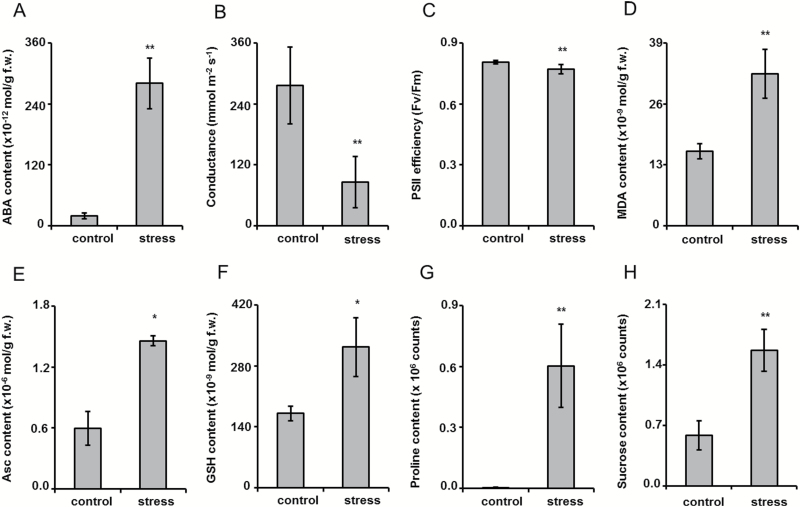
Characterization of plant stress developed at 3 d after transfer of Arabidopsis plants on agar medium saturated with PEG-free (control) and PEG 8000 solutions with ψ=–0.4 MPa (172.27 g l^–1^) by the tissue contents of abscisic acid (A), stomatal conductance (B), PS II efficiency (C), malondialdehyde content (D), ascorbic acid content (E), reduced glutathione content (F), proline content (G), and sucrose content (H). ** and * denote statistical significance at the confidence levels *P*<0.01 and *P*<0.05, respectively.

The observed plant dehydration was accompanied by a 14.3-fold increase of abscisic acid (ABA) content (*t*-test, *P*=0.001, Fig. 3A), stomata closure (Fig. 3B, *t*-test, *P*=1.1 × 10^–5^), and a reduction of photosystem II (PSII) efficiency (*t*-test, *P*=7.1 × 10^–4^, Fig. 3C), whereas the leaf relative water content (LRWC) and chlorophyll contents were unchanged (Supplementary Fig. S1A, B). On the biochemical level, the osmotic stress was manifested with an increase in the contents of thiobarbituric acid-reactive substances, expressed as malondialdehyde (MDA) equivalents (Fig. 3D, *t*-test, *P*=0.006). It was accompanied by the approximately 2.5-fold increase in ascorbic acid (Asc; *t*-test, *P*=0.02) and about a 2-fold increase (*t*-test, *P*=0.036) in GSH amounts (Fig. 2E and F, respectively), whereas the abundances of their oxidized counterparts (dehydroascorbic acid (DHA) and GSSH) did not show changes (Supplementary Fig. S1C, D). Nevertheless, the Asc/DHA and GSH/GSSG ratios were unchanged (Supplementary Fig. S1E, F), which indicated preserved antioxidant capacity of the ascorbate–glutathione cycle. Additionally, development of stress was confirmed by the accumulation of osmolytes: a 152- and 2.7-fold abundance increase was registered for proline (*t*-test, *P*=0.007) and sucrose (*t*-test, *P*=0.004), respectively (Fig. 3G, H).

**Fig. 4.**
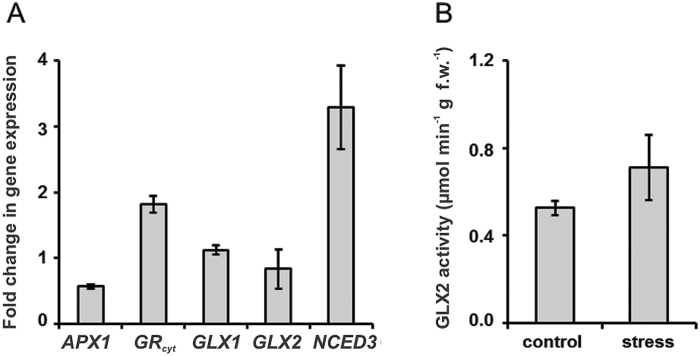
Relative expression levels of cytosolic *ascorbate peroxidase 1* (*APX1*, *At1g07890*), cytosolic *glutathione reductase* (*GRcyt*, *At3g24170*), *glyoxalase I (GLX1*, *At1g08110*), *glyoxalase II* (*GLX2*, *At2g43430*), and *9-cis-epoxycarotenoid dioxygenase 3* (*NCED3*, *At3g14440*) genes determined by RT-qPCR analysis (A) and activity of glyoxalase II in the leaves of Arabidopsis plants grown for 3 d on agar medium saturated with PEG-free (control) and PEG 8000 solutions with ψ=–0.4 MPa (172.27 g l^–1^) (B).

The gas chromatography–mass spectrometry (GC-MS)-based profiling of primary metabolites revealed an increase in leaf tissue contents of other (besides proline) amino acids and some intermediates of the tricarboxylic acid cycle (Supplementary Fig. S2). Moreover, multiple sugars, also known to be potent glycation agents (e.g. arabinose, ribose, sugar phosphates) were clearly up-regulated (Supplementary Fig. S3). However, surprisingly, the levels of α-dicarbonyls (glyoxal and methylglyoxal) remained unchanged (Supplementary Fig. S4). The RT-qPCR analysis revealed a two-fold decrease of the cytosolic *ascorbate peroxidase 1* (*APX1*, *At1g07890*) transcript abundance, whereas the transcripts of cytosolic *glutathione reductase* (*GRcyt*, *At3g24170*) and *9-cis-epoxycarotenoid dioxygenase 3* (*NCED3*, *At3g14440*) were up-regulated two- and three-fold, respectively (Fig. 4A). The expression levels of *glyoxalase I* and *II* (*GLX1*, *At1g08110* and *GLX2*, *At2g43430*) were not affected by the stress conditions, although the hydrolase activity of the corresponding *GLX2* product was slightly increased (0.71 *vs* 0.53 μmol min^−1^ g f.w.^−1^ in controls, Fig. 4B).

### Protein isolation and tryptic digestion

To cover all changes in the AGE proteome, the total protein fraction was extracted from ground leaf material. For this, a phenol extraction protocol was selected as the most effective and straightforward approach (Isaacson *et al.*, 2006). Therefore, each sample was isolated from two portions of tissue (approximately 250 mg each). The dried protein residues were combined after reconstitution. The protein concentrations and yields obtained were in the range of 1.5–3.7 mg ml^–1^ and 0.7–1.5 mg g^–1^ fresh weight, respectively (Supplementary Table S7). The precision of the protein determination was confirmed by SDS-PAGE. The UV densities at 595 nm (obtained by the Gel Doc^TM^ EZ Imager equipped with Image Lab software; Bio-Rad, München, Germany) were 2.40 × 10^6^±5.31 × 10^5^ arbitrary units (relative standard deviation: 22%), thereby confirming sufficient precision of the Bradford assay (Supplementary Fig. S5, Supplementary Table S7). The following tryptic digestion was considered to be complete, when the RuBisCO large subunit band was not detectable any more (Supplementary Fig. S6), indicating a digestion efficiency better than 95% (assuming the sensitivity of the staining is better than 30 ng (Frolov *et al.*, 2014*a*) and RuBisCO content of at least 20% of the total leaf protein (Cellar *et al.*, 2008)).

### Protein quantification

Overall 351 differentially expressed proteins could be quantified by at least two unique unmodified peptides (Supplementary Table S8). Among these, 80 were significantly regulated (*P*<0.05) within the control groups: 25 proteins showed higher abundances in the stress group, whereas 55 proteins were significantly more highly expressed in the control group.

### Identification of AGE-modified sites in proteins

**Fig. 5.**
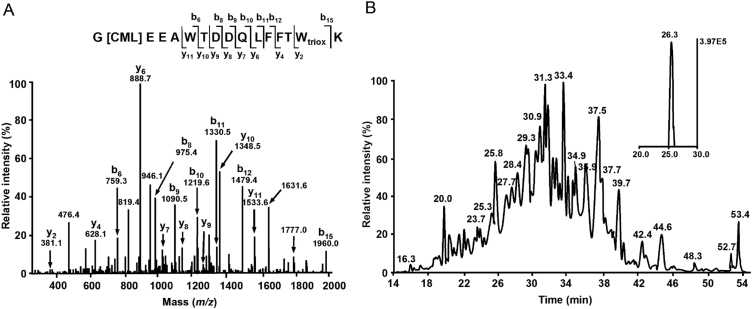
Tandem mass spectrum of the *m*/*z* 1053.96 corresponding to [M+2H]^2+^ of the tryptic peptide G[CML]EEAWTDDQLFFTW_triox_K, representing acetyl-CoA carboxylase 2 (A), and annotation of this peptide by its characteristic *t*_R_ in the corresponding XIC at *m*/*z* 1053.96 ± 0.02 (B).

**Fig. 6.**
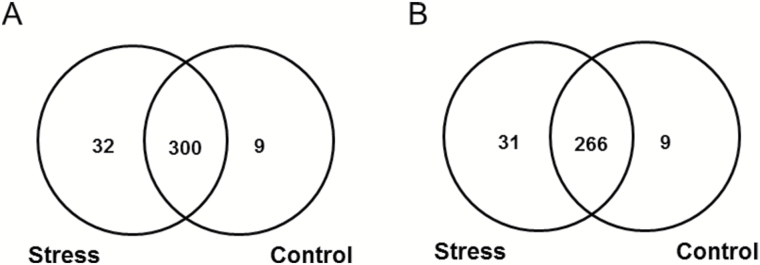
Numbers of AGE-modified peptides (A) and proteins (B) annotated by tandem mass spectrometry (MS/MS) fragmentation patterns verified for *t*_R_ and *m*/*z* of the corresponding peptide signals in all samples.

On the basis of all data-dependent acquisition (DDA) experiments, altogether 785 AGE-containing peptides (representing 724 proteins) could be annotated with a high confidence (XCorr≥2.20 for doubly and 3.75 for highly charged peptides) in both treatment groups. The localization of AGE-modified amino acids could be unambiguously identified by characteristic series of b- and y-ions observed in the corresponding tandem mass spectra (Fig. 5A). After the cross-annotation of identified peptides in all samples by *t*_R_ and *m*/*z* of the corresponding quasi-molecular ions (Fig. 5B), the majority of AGE peptides (797 hits) were common for both groups, while only 33 and 62 species were unique for control and stressed plants, respectively (in total 855 proteins). Although all glycated peptides were identified with a high confidence, identification of proteins must rely at least on two peptides (Carr *et al.*, 2014). Thus, annotation of a second peptide (besides an AGE-modified one) was necessary to identify each protein. To identify the second peptides for all 855 proteins, the raw data representing individual HILIC fractions, acquired in three gas phase fractionation segments without application of exclusion lists, were searched against a FASTA file (generated by DNA and protein sequence alignment software) containing sequence information of the glycated proteins. The search revealed altogether 306 proteins represented by 341 AGE-modified peptides (Fig. 6). In the stress-treated group, 297 proteins were annotated, 31 of which were stress-specific, whereas the control group comprised 275 glycated proteins. The pattern of AGE-modified peptides found in control group is summarized in Supplementary Table S9.

**Fig. 7.**
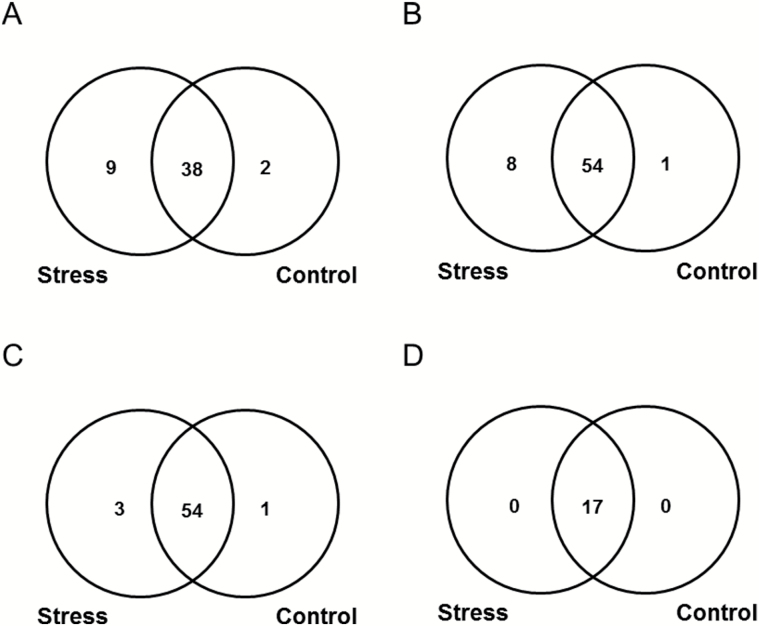
Numbers of modified peptides representing specific AGE classes identified by MS/MS fragmentation patterns in drought-treated and control groups: Glarg (A), CMA/GD-HI (B), CEL (C) and pyrraline (D).

The pattern of individual AGE classes was dominated by CML, CMA (or isomeric glyoxal-derived dihydroxyimidazolidine, GD-HI (Frolov *et al.*, 2014*b*) and MG-H (Supplementary Fig. S7). To assess the effect of osmotic stress on the patterns of individual AGEs, we considered the number of the modified tryptic peptides, representing corresponding structures. Surprisingly, a differential accumulation of individual AGE classes was observed. Thus, the plants subjected to osmotic stress showed essentially higher absolute numbers of unique peptides containing Glarg and CMA/GD-HI residues (Fig. 7A, B). To a lesser extent, stress-related increases in the number of modification sites were observed for CEL, GLAP, MG-H, CML, and CEA or methylglyoxal-derived dihydroxyimidazolidine (MGD-HI) (Fig. 7C and Supplementary Fig. S8C, D, E and F, respectively). In contrast to the former AGE types, the patterns of pyrraline, Argpyr and TH-Pyr were not affected by osmotic stress (Fig. 7D and Supplementary Fig. S8A and B, respectively).

**Fig. 8.**
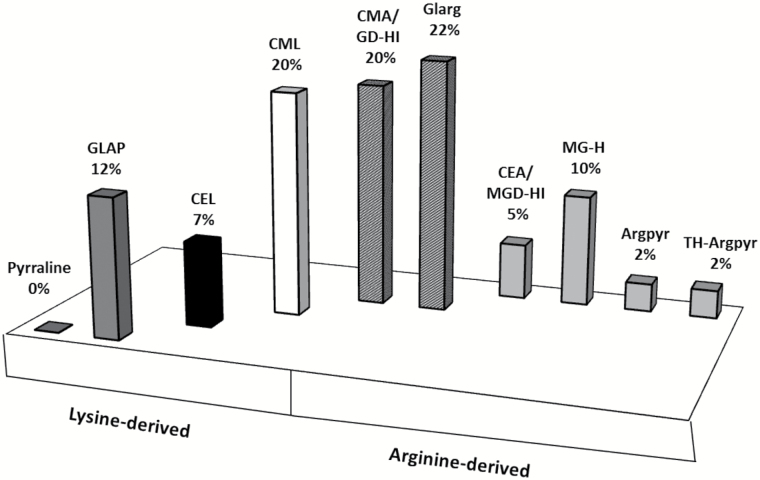
Relative modification rates and metabolic origin of individual AGE classes in stress-specific unique tryptic peptides obtained from the total protein isolated from the leaves of drought-treated Arabidopsis plants. Shading indicates origin of AGEs: dark grey, sugar/early glycation end-products;black, sugar/early glycation products/MGO; white, sugar/early glycation end-products/GO; dark grey with white marks, GO; light grey, MGO.

The group of unique stress-specific peptides comprised 32 peptides, representing 31 proteins with totally 41 AGE sites (Table 1), and was dominated by three specific modifications, namely Glarg, CML and CMA/GD-HI. All these modifications represented more than 62% of the unique modified residues and originated metabolically from the same carbonyl compound, glyoxal, or, like CML, partly from free sugars and early glycation products (Ferreira *et al.*, 2003) (Fig. 8). Surprisingly, each of the individual methylglyoxal-derived modifications (CEA/MGD-HI, MG-H, Argpyr and TH-Pyr) represented only 2–10% of the total stress-specifically modified residues and comprised only one-fifth of modified residues in total. The relative levels of CEL were low (7%), though this modification could also, at least partially, originate from sugars and Amadori or Heyns compounds (Nagai *et al.*, 2003). The modifications originating solely from free sugars and their degradation products (pyrraline and GLAP) were less abundant as compared with those from precursors such as glyoxal and methylglyoxal (Fig. 8). The functional annotation of the stress-specific proteins revealed 14 functional classes, dominated by proteins involved in regulation of transcription with four entries, followed by three entries each of proteins involved in photosynthesis, lipid metabolism and protein metabolism. The functional annotation for seven entries was not assigned (Table 1, Supplementary Fig. S9).

**Table 1.** Unique stress-specific AGE-modified peptides identified in the tryptic digests obtained from the Arabidopsis plants grown for three days on the agar medium infused with 172.27 g l^–1^ PEG 8000

**Nr.**	**Peptide sequence**	***m*/*z***	***z***	**XCorr**	***t***_**R**_	**Protein annotation**		
						**Site**	**Accession number**	**Protein name** ^***a***^
1	QEVY[MG-H]MPSVGCISAVLKK[CEA]	1174.12	2	2.2	1.85	R_1904_, R_1918_	Q5IBC5	Separase^1^
2	MLW[CMA]CNWVKQRK	868.42	2	2.2	16.99	R_4_	Q3EDA9	Putative pentatricopeptide repeat-containing protein At1g16830^2^
3	[MG-H]MNT[CEL]NVSAWNMKR	939.47	2	2.2	19.65	R_508_, K_512_	Q9LH74	Mechanosensitive ion channel protein 5^2^
4	H[GLAP]AEETATMNTTRVAVGK	1035.02	2	2.5	17.45	K_863_	Q9SB63	Protein MODIFIER OF SNC1 1^2^
5	[CEL]MDRAIEYLERMVS[CMA]	1022	2	2.3	22.68	K_394_, R_408_	Q3EDF8	Pentatricopeptide repeat-containing protein At1g09900^2^
6	CNPSVLWCYKDKLDISSH[CML]QK	1307.61	2	2.3	28.65	K_68_	Q9M2Q4	RNA cytidine acetyltransferase 2^2^
7	FGKVGADWAY[GLAP]GMREK	1038.5	2	2.5	32.5	K_564_	Q9LER0	Pentatricopeptide repeat-containing protein At5g14770, mitochondrial^2^
8	IEESLELINDMVTRGYLP[Argpyr]	1164.59	2	2.5	35.89	R_527_	Q84VG6	Pentatricopeptide repeat-containing protein At2g17525, mitochondrial^2^
9	TGTLKPGPCVMKISP[CML]	865.97	2	2.2	18.37	K_372_	Q9M3B6	Plastidial pyruvate kinase 4, chloroplastic^3^
10	QQLLSEIS[Glarg]LMNKYK	954.01	2	2.3	18.56	R_279_	Q9SIN9	Phospholipase A1-Ialpha2, chloroplastic^3^
11	MLDFDFLCG[CMA]	637.79	2	2.4	34.01	R_35_	Q9C522	ATP-citrate synthase beta chain protein 1^3^
12	MKEFAE[CMA]LGW[MG-H]MQK	969.47	2	2.2	19.11	R_202_, R_206_	Q9SVL0	Zinc-finger homeodomain protein 7^4^
13	E[CMA]AVYKCSCGKVK	829.91	2	2.5	21.36	R_270_	Q9FFK8	NF-X1-type zinc finger protein NFXL2^4^
14	N[GLAP]EAEAGTSKSSGDAEQSSK	1060.48	2	2.3	23.76	K_309_	Q9SK74	Zinc finger CCCH domain-containing protein 21^4^
15	ELQV[Glarg][Glarg]FMFDCVEGK	984.95	2	2.3	32.19	R_112_, R_113_	Q7XJK5	Agamous-like MADS-box protein AGL90^4^
16	MVAPCWR[CMA]PSVK	782.89	2	2.5	19.59	R_17_	Q9LHJ9	Probable protein phosphatase 2C 38^5^
17	[Glarg]PQGLYISL[CEL]EK	780.42	2	2.3	19.6	R_1_, K_10_	Q9XGZ0	NADP-dependent malic enzyme 3^6^
18	HVLSFARFTH[TH-Pyr]YGKK	1050.56	2	2.2	20.44	R_65_	Q8H166	Thiol protease aleurain^7^
19	ELSITDLSPSIAL[CMA]	786.92	2	2.3	30.54	R_90_	Q9SFX2	U-box domain-containing protein 43^7^
20	MGTNALVPGFEMGI[MG-H]	839.91	2	2.2	33.38	R_183_	Q0WRJ7	Peptidyl-prolyl cis-trans isomerase FKBP20-2, chloroplastic^7^
21	SG[Glarg]TGRAGNTGVAVTLYDSRK	1110.57	2	2.4	21.07	R_436_	Q39189	DEAD-box ATP-dependent RNA helicase 7^8^
22	LWDL[CEA][CML]LR	615.35	2	2.2	35.34	R_424_, K_425_	O22785	Pre-mRNA-processing factor 19 homolog 2^8^
23	G[Glarg] GGSTGYDNAVALPAGGRGDEEELVKENVK	801.14	4	4.1	24.33	R_247_	P23321	Oxygen-evolving enhancer protein 1-1, chloroplastic^9^
24	SQAETGEIKGHYLNATAGTCEEMI[CML]	924.42	3	4	24.76	K_252_	O03042	Ribulose bisphosphate carboxylase large chain^9^
25	LPLFGCTDSAQVL[GLAP]EVEEC[GLAP]	828.73	3	4.4	30.38	K_140_, K_146_	P10795	Ribulose bisphosphate carboxylase small chain 1A, chloroplastic^9^
26	WSPELAAACEVW[CML]	790.35	2	2.5	32.89	K_463_	O03042	Ribulose bisphosphate carboxylase large chain^9^
27	ELCGRVVGSDCKIEGT[CML]	954.97	2	2.2	24.46	K_823_	Q9LYN8	Leucine-rich repeat receptor protein kinase EMS1^10^
28	MDKKTIVWF[Glarg]R	778.41	2	2.3	25.6	R_12_	Q96524	Cryptochrome-2^10^
29	KSNIWISD[Glarg]NPDSRR	958.47	2	2.2	27.41	R_152_	Q9ZT82	Callose synthase 12^11^
30	TLQALQYIQENPDEVCPAGW[CML]PGEK	968.79	3	4.5	30.41	K_246_	Q96291	2-Cys peroxiredoxin BAS1, chloroplastic^12^
31	GYFAWCLGDNYELWPS[Glarg]	1101.46	2	2.3	31.48	R_416_	Q3E8E5	Putative myrosinase 3^13^
32	NLNG[CMA]DGMKW[CML]DFR	926.95	2	2.2	35.54	R_511_, K_517_	Q9STT6	ABC transporter A family member 6^14^

^*a*^The peptides are listed in order of protein functional groups: ^1^cell division and cell cycle; ^2^unclassified, no ontology and unknown; ^3^lipid metabolism; ^4^regulation of transcription; ^5^protein modification; ^6^energy metabolism; ^7^protein metabolism; ^8^RNA metabolism; ^9^photosynthesis; ^10^regulation/signaling; ^11^cell wall biosynthesis; ^12^redox; ^13^enzyme families; ^14^transport.

### Quantification of AGE-modified tryptic peptides

In the next step, we performed label-free quantification of the AGE-modified peptides identified in both treatment groups (Fig. 6A). All 300 AGE-modified peptides, annotated in both stress and control plants, could be successfully integrated in the Orbitrap-MS scans. For these species, extracted ion chromatograms (XICs) (*m*/*z*±0.02) were obtained and peak integration at the specific *t*_R_ was performed (Fig. 5B). As could be judged from the peak areas integrated in corresponding XICs, the abundance of 12 glycated peptides (representing 15 AGE-modified sites in 12 proteins) changed upon the application of osmotic stress. Among the identified differentially glycated sites, 15 were affected significantly (*t*-test *P*<0.05). These sites were represented by 12 peptides originating from 12 proteins. Thus, the abundances were decreased (1.5–2.5-fold) and increased (1.4–27.5-fold) for eight and four AGE-containing peptides, respectively (Supplementary Table S10). The functional annotation of these proteins revealed 10 groups, dominated by those responsible for regulation of lipid metabolism and involved in protein ubiquitination and degradation represented by two proteins each (Supplementary Table S10, Supplementary Fig. S10).

### Protein carbonylation

The quantification analysis relied on density of immunofluorescence of whole sample lines. The fluorescence intensity observed in the control and stressed plants was 14.8 × 10^6^±2.5 × 10^5^ and 22.3 × 10^6^±3.0 × 10^5^, respectively. Thus drought conditions promoted significantly (1.5-fold, *t*-test *P*=0.029) protein carbonylation (Supplementary Fig. S11).

## Discussion

### Drought establishment and stress response

Long-term withholding of water, applied to plants grown in soil, is a common experimental strategy used in drought stress research (Koffler *et al.*, 2014). Under these conditions, the water potential (ψ_w_) of the soil is gradually reduced in the course of experiments. Although the rates of soil drying differ from pot to pot, this variability might not affect reproducibility over long periods. However, the study of short-term drought responses needs to rely on constant ψ_w_, as only this can provide reproducible and well-interpretable results. In this context, growth of plants in polyethylene glycol (PEG)-containing medium is the method of choice, as this substance does not penetrate plant cells, and does not cause plasmolysis (in contrast to mannitol and sorbitol) (Verslues *et al.*, 2006). Even more advantageous is the use of PEG-saturated agar as a medium: it relies on a solid substrate with constant ψ_w_ (van der Weele *et al.*, 2000). Moreover, due to partial immobilization of PEG molecules in the three-dimensional agar net, in this model, any negative effect of PEG on root epithelium (which might also impact drought symptoms) is excluded. Thus, to address specifically the early drought-related effects on the plant glycated proteome, we selected this model of controlled dehydration.

Although we did not observe the reduction of the leaf ψ_w_ on the third day of the experiment, it could be clearly seen when the plants were grown on dehydrated medium for at least 1 week (Supplementary Fig. S12A). Hence, the reduction of leaf ψ_w_ occurs later than the primary physiological and biochemical changes. This confirms the correctness of our approach, aiming at the registration of the short-term drought-related glycation proteome responses. It is also supported by the dramatic increase in ABA contents, stomata closure, and decrease in PSII efficiency (Fig. 3A–C) that are characteristic for the early plant drought response (Liu *et al.*, 2016). In this context, the absence of a decrease in chlorophyll content can be explained by its expected later onset, as photosynthesis is only barely affected under the conditions of mild and moderate stress (Harb *et al.*, 2010). The same is valid for LRWC, as was shown in our additional 7-day-long drought experiment (Fig. S12B). On the biochemical level, mobilization of the anti-oxidative defense (i.e. up-regulation of Asc and GSH; Fig. 3E, F) with unchanged Asc/DHA and GSH/GSSG ratios (Supplementary Fig. S1E, F) was in accordance with the physiological data and increase of the oxidative stress markers (Bou *et al.*, 2008), i.e. thiobarbituric acid-reactive substances (Fig. 3D and Supplementary Fig. S1G, H). Principal component analysis (PCA) revealed clear differences between control and stressed plants at the metabolome level (Supplementary Fig. S13). Moreover, as was revealed by means of the linear regression models built on the base of MATLab 2016A (http://www.mathworks.com/), the abundances of thiobarbituric acid-reactive substances and GSH showed significant correlation with those of sugar-related metabolites (*R* ^2^=0.802, *P*=0.00994 and *R* ^2^=0.808, *P*=0.00935, respectively, see Supplementary Calculations S1).

### Characterization of the AGE-modified proteome

Although the methods to analyze plant glycoproteins are well established (Song *et al.*, 2013), the approaches for characterization of plant glycated proteome were proposed only recently (Bilova *et al.*, 2016). However, analysis of AGEs (in most cases structurally not related to carbohydrates) cannot rely on regular enrichment procedures, conventional for glycoproteomics (Zhang *et al.*, 2011). Because of the high heterogeneity of AGE structures, antibody-based two-dimensional gel electrophoresis (2D-GE) techniques are hardly applicable to analysis of advanced glycation, as western blotting conditions need to be optimized and validated for multiple primary antibodies of mostly unknown specificity. Hence, application of the liquid chromatography-based techniques is desired for AGE analysis. However, due to plenty of existing AGE structures and extremely low relative abundances of individual modified peptides, the only way to overcome the so-called undersampling effect and to increase the rates of AGE discovery is implementation of fractionation techniques. Therefore, here we combined in one analytical workflow pre-fractionation on the level of chromatographic separation (Bollineni *et al.*, 2011), and gas phase fractionation in combination with so-called exclusion lists in the step of MS analysis (Frolov *et al.*, 2014*a*). To the best of our knowledge, this methodological platform was applied in glycation research for the first time, although pre-fractionation with HILIC proved to be an effective tool in the study of protein carbonylation and phosphorylation in application to human proteomics (Singer *et al.*, 2010; Bollineni *et al.*, 2011).

After the *t*_R_- and *m*/*z*-based verification of DDA results, 32 AGE-modified peptides (i.e. approximately 10% of the total detected number in the group subjected to osmotic stress) could be considered as unique (i.e. not detected in controls). Therefore, it cannot be excluded that the patterns of the stress-specific peptides would change when the analysis is performed with more sensitive mass analyzers (e.g. modern QqTOF instruments). Functional annotation of the stress-specific proteins revealed 14 groups of polypeptides potentially damaged by AGE modifications under drought stress conditions (Table 1, Supplementary Fig. S9). Some of these proteins were reported earlier to be affected by environmental stress. Their modified state, proved here, can, at least partly, underlie these changes by regulatory effects and enhancement of protein catabolism upon non-enzymatic protein modifications. Thus, the stress-specific carboxymethylation of acetyl-CoA carboxylase observed here (i.e. one of the major lipid biosynthesis enzymes, Supplementary Table S10) was in accordance with the up-regulation of this protein under drought conditions (Kottapalli *et al.*, 2009). Detection of analogously modified ABC transporter (Table 1) can be underlain by its up-regulation as well (Kang *et al.*, 2011). It is known that loss of the NFX1-type zinc finger protein (NFXL2) gene results in elevated ABA levels, reduced stomatal conductance, and enhanced drought tolerance (Lisso *et al.*, 2011). The advanced glycation at R_320_ (CMA) observed here could represent the natural mechanism impacting in the ABA-dependent regulatory events.

**Fig. 9.**
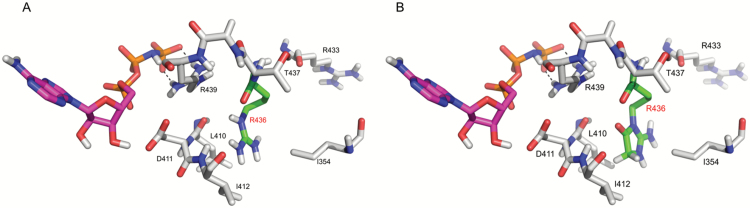
DEAD-box ATP-dependent RNA helicase without (A) and with (B) glyoxal-derived hydroimidazolone (Glarg) modification of arginine 436 leading to its conformational change. Effects of this change on the binding of ATP (magenta carbon atoms) cannot be excluded.

Obviously, the structural changes, related to advanced glycation, might affect functional state and activity of proteins. To address the possibility of these effects, we performed homology modeling for the stress-specifically glycated proteins (Supplementary Protocol S8, Supplementary Table S11). We find this approach advantageous in comparison to enzymatic activity tests, as in the latter case the structure-related effects of glycation can be hardly distinguished from inhibition and allosteric regulatory events. Moreover, it is necessary to take into account the fact that glycation occurs simultaneously at multiple sites, which prevents synthesis of standards with the desired glycation status. Evaluation of the resulting models revealed mostly surface localization of glycation sites. Obviously, it can directly affect assembly and integrity of oligomeric proteins, which according to our results can be the case for RuBisCO, both small and large subunits of which are surface glycated (Table 1). Indeed, earlier, Yamauchi and co-workers described *in vitro* inactivation of this enzyme in the presence of glycation agents (Yamauchi *et al.*, 2002). The carboxymethylation at K_13_ and K_25_ observed here respectively for two different peptides might contribute to partial *in vivo* RuBisCO inactivation. Further, the proteins interacting with nucleic acids were typically glycated by arginines of DNA/RNA-binding domains (e.g. the homeobox domain of the zinc finger homeodomain protein 7 or coiled-coil domain of the agamous-like MADS-box protein). This fact was in accordance with previous observations (Thornalley, 2003). Glycation of enzymes can directly affect substrate- or metal-binding centers. For example, glycation of the arginine 436 in the DEAD-box ATP-dependent RNA helicase (Fig. 9) leads to a conformational change of this residue. Therefore, it cannot be excluded that in the active site the binding of ATP (magenta carbon atoms) will also be influenced due to backbone conformational alterations. Analogously, in myrosinase 3, glycation of R416 might affect binding of substrate (residues 411 and 412). Similarly, glycation was observed in the catalytic domains of cryptochrome-2 (photolyase domain) and thioredoxin domain of 2-cys peroxiredoxin BAS 1.

Remarkably, only seven AGE classes (among those considered here) demonstrated higher numbers of stress-specific modification sites (Fig. 7A–C and Supplementary Fig. S8C–F). The most striking differences were observed for the peptides containing the two glyoxal-derived residues: Glarg and its hydrolysis product, CMA. It is important to mention that the increment of 58 *m*/*z* can also correspond to GD-HI, known to be the Glarg precursor (Frolov *et al.*, 2014*b*). In contrast, the differences in the modification rates were less pronounced for the methylglyoxal-derived modifications (MG-H and CEA), which was in accordance with higher abundance of glyoxal in leaf tissues (Supplementary Fig. S4). Lower abundances of methylglyoxal can be explained by the high levels of antioxidants and carbonyl scavengers, as well as high or specific activity of the glyoxalase system, responsible for detoxication of α-dicarbonyls, where GSH plays the central role (Yadav *et al.*, 2008; Gill and Tuteja, 2010). Finally, the modifications originating solely from sugars and their degradation products (GLAP and pyrraline) were the least abundant. The number of GLAP-modified sites was increased by stress while pyrraline sites were left unaffected. These data were confirmed by the origin of the stress-specific AGE-modified peptides: 62% of the species at least partly originated from glyoxal (Fig. 8). This clearly indicates that glyoxal is the main intermediate of AGE formation under drought conditions. As the levels of individual carbohydrates were increased upon 3 d of stress application (Supplementary Fig. S3), it is logical to assume that sugar autoxidation might be the primary source of glyoxal (Thornalley *et al.*, 1999), although lipid peroxidation cannot be excluded as well. These enhanced levels of monosaccharide autoxidation can be explained by accumulation of sugars in the presence of transition metal ions in parallel to the development of oxidative stress (Wolff and Dean, 1987). Thus, up-regulation of galactinol, glucose-6-phosphate, and dihydroxyacetone phosphate might be the reason for enhanced glycation of plant proteins (Supplementary Fig. S3), which is in agreement with the high reactivity of these sugars in model peptide-based glycation systems (Bilova *et al.*, 2016). This fact was additionally confirmed by a strong correlation in abundance changes observed for these sugars and malondialdehyde equivalents (see Supplementary Calculations S1).

Surprisingly, the leaf contents of α-dicarbonyl products (glyoxal and methylglyoxal) characteristic for this pathway were slightly decreased (Supplementary Fig. S4). Based on our data, this can be explained by several factors: (i) the strong up-regulation of amino acid biosynthesis (Supplementary Fig. S2) and enhancement of α-dicarbonyl scavenging, (ii) relatively high contents of GSH (Fig. 3F), (iii) increase of α-dicarbonyl detoxification via glyoxalase system (Fig. 4B), and (iv) enhanced involvement of α-dicarbonyl in reactions with amino acid side chains of proteins with formation of AGEs (Figs 6 and 7A, B). The latter assumption was also confirmed by statistically significant increases in the degree of protein carbonylation under the drought conditions (Supplementary Fig. S11).

Thus, our results provide the proteomic and metabolomic support for the results of Bechtold and co-workers, obtained on the amino acid level by exhaustive enzymatic protein hydrolysis (Bechtold *et al.*, 2009). The observation of only a few pyrraline modification sites supports the assumption about the minor importance of the early glycation pathway in AGE formation. This is in contrast to mammalian systems, where osone-, glyoxal-, and methylglyoxal-derived AGEs are present in comparable abundances (Ahmed *et al.*, 2005).

## Conclusions

Due to the pronounced pro-inflammatory effect of AGEs in the human organism (Lennicke *et al.*, 2016), consumption of these products might impact the development of systemic sub-clinical inflammation and related diseases, like atherosclerosis and diabetes mellitus. Thus, accumulation of AGEs during food processing and growth of crop plants in the field ideally has to be avoided. In this study we demonstrate that even a short-term application of osmotic stress (which corresponds well to agricultural practice) clearly enhances the rate of protein glycation in Arabidopsis: 43 AGE-modified proteins (i.e. 14% of the total AGE-containing proteome) were affected by this environmental factor. Obviously, longer exposure to osmotic stress might increase this protein damage, potentially harmful for mammals and with yet unknown consequences for the plants themselves. This aspect needs to be addressed by long-term stress experiments, supported with further functional assays. This also needs to be considered when new strategies to increase the drought tolerance in plants are developed. In this context, prospective anti-glycation agents (e.g. scavengers of reactive carbonyls) can be applied to protect plant proteins against advanced glycation.

## Supplementary data

Supplementary data are available at *JXB* online.

Protocol S1. Determination of lipid hydroperoxide contents.

Protocol S2. Determination of hydrogen peroxide contents.

Protocol S3. Determination of malondialdehyde (MDA) contents.

Protocol S4. Determination of ascorbic and dehydroascorbic acid contents.

Protocol S5. Gene expression analysis.

Protocol S6. Determination of protein concentrations by the Bradford assay.

Protocol S7. Sodium dodecyl sulfate-polyacrylamide gel electrophoresis (SDS-PAGE).

Protocol S8. Protein homology modeling.

Calculations S1. Linear regression analysis

Fig. S1. Characterization of stress parameters in *A. thaliana* plants.

Fig. S2. Relative abundances of organic acids in *A. thaliana* leaf tissues.

Fig. S3. Relative abundances of carbohydrates in *A. thaliana* leaf tissues.

Fig. S4. The contents of free glyoxal and methylglyoxal in *A. thaliana* leaf tissues.

Fig. S5. SDS-PAGE of individual *A. thaliana* leaf protein isolates.

Fig. S6. SDS-PAGE of individual *A. thaliana* leaf protein tryptic digests.

Fig. S7. Distribution of AGEs by classes in the proteins obtained from control *A. thaliana* plants.

Fig. S8. Numbers of modified peptides representing specific AGE classes identified by MS/MS fragmentation patterns in *A. thaliana* drought-treated and control plants.

Fig. S9. Functional annotation of the unique osmotic stress-specifically AGE-modified *A. thaliana* proteins.

Fig. S10. Functional annotation of the AGE-modified *A. thaliana* proteins demonstrating significantly (*P*≤0.05) different abundances (in comparison with controls) of corresponding glycation sites.

Fig. S11. Anti-dinitrophenylhydrazine western blot analysis for detection of protein carbonylation.

Fig. S12. The water potentials and leaf relative water contents of *A. thaliana* plants grown for three and seven days in presence and absence of PEG-induced osmotic stress

Fig. S13. **Principal component analysis (PCA**) of the primary metabolites and drought stress markers.

Table S1. Primer sequences for target and reference genes used in RT-qPCR assays.

Table S2. GC separation conditions and EI-Q-MS settings for metabolite analysis.

Table S3. Parameters of the HILIC separation method.

Table S4. Reconstitution of HILIC fractions for nanoUPLC-MS/MS experiments.

Table S5. Parameters of the nanoUPLC separation method.

Table S6. Instrument settings applied for ESI-Orbitrap-LIT-MS experiments.

Table S7. Protein recoveries and total UV densities calculated for individual samples separated by SDS-PAGE.

Table S8. Differentially abundant proteins related to the osmotic stress in *A. thaliana* leaf.

Table S9. Glycation sites detected in control *A. thaliana* plants.

Table S10. Glycation sites in *A. thaliana* plants affected significantly by PEG osmotic stress.

Table S11. Summary of protein homology modeling performed for the stress-specifically AGE-modified proteins peptides.

Supplementary Data
