# Supplementary material for: Osmotic stress is accompanied by protein glycation in Arabidopsis thaliana
Source: J Exp Bot. 2016 Nov 10;67(22):6283–95. doi: 10.1093/jxb/erw395 (PMC5181577; doi:10.1093/jxb/erw395)
Supplement: Supplementary Data [file supp_67_22_6283__index.html]

Osmotic stress is accompanied by protein glycation in Arabidopsis thaliana — Osmotic stress is accompanied by protein glycation in Arabidopsis thaliana — Supplementary Data 

# Osmotic stress is accompanied by protein glycation in *Arabidopsis thaliana*

## Supplementary Data

Data files

- supplementary\_protocols\_S1\_S8\_figures\_S1\_S13\_Tables\_S1\_S7\_S10\_S11.pdf - Supplementary Data
- supplementary\_table\_S8.xls - Supplementary Data
- supplementary\_table\_S9.xls - Supplementary Data
