# Supplementary material for: Osmotic stress is accompanied by protein glycation in Arabidopsis thaliana
Source: J Exp Bot. 2016 Nov 10;67(22):6283–95. doi: 10.1093/jxb/erw395 (PMC5181577; doi:10.1093/jxb/erw395)
Supplement: Supplementary Data [file supp_erw395_supplementary_protocols_S1_S8_figures_S1_S13_Tables_S1_S7_S10_S11.pdf]

Changes in *Arabidopsis thaliana* advanced glycated proteome induced by the polyethylene glycol-related osmotic stress

Gagan Paudel,[1,2†] Tatiana Bilova,[1,2,3†] Rico Schmidt,[4] Uta Greifenhagen,[2] Robert Berger,[1] Elena Tarakhovskaya,[3] Stefanie Stöckhardt,[5] Gerd Ulrich Balcke,[6] Klaus Humbeck,[5] Wolfgang Brandt,[1] Andrea Sinz,[4] Thomas Vogt,[6] Claudia Birkemeyer,[2] Ludger Wessjohann[1] and Andrej Frolov[1,2*]

Supplementary data 1

[1]Department of Bioorganic Chemistry, Leibniz Institute of Plant Biochemistry; [2]Faculty of Chemistry and Mineralogy, Universität Leipzig; [3]Department of Plant Physiology and Biochemistry, St. Petersburg State University; [4]Department of Pharmaceutical Chemistry and Bioanalytics, Institute of Pharmacy, Martin-Luther Universität Halle-Wittenberg; [5]Department of Plant Physiology, Martin-Luther Universität Halle-Wittenberg; [6]Department of Metabolic and Cell Biology, Leibniz Institute of Plant Biochemistry

[†]These authors contributed equally to the manuscript

*Corresponding author:
Dr. Andrej Frolov
Leibniz Institute of Plant Biochemistry
Department of Bioorganic Chemistry
Weinberg 3, 06120, Halle/Saale, Germany
Tel. +49 (0) 345 55821370
Fax. +49 (0) 345 55821309
Email: afrolov@ipb-halle.de

Directory

Protocol S1 Determination of lipid hydroperoxides S-4

Protocol S2 Determination of hydrogen peroxide S-5

Protocol S3 Determination of malondialdehyde (MDA) contents.......................................S-6

Protocol S4 Determination of ascorbic and dehydroascorbic acid contents S-7

Protocol S5 Gene expression analysis S-8

Protocol S6 Determination of protein concentrations by the Bradford assay.......................S-9

Protocol S7 Sodium dodecyl sulfate-polyacrylamide gel electrophoresis (SDS-PAGE)...S-10

Protocol S8 Protein homology modeling..S-11

Table S1 Primer sequences for target and reference genes used in RT-qPCR assays S-12

Table S2 GC separation conditions and EI-Q-MS settings for metabolite analysis S-13

Table S3 Parameters of the HILIC separation method S-14

Table S4 Reconstitution of HILIC fractions for nanoUPLC-MS/MS experiments............S-15

Table S5 Parameters of the nanoUPLC separation method S-16

Table S6 Instrument settings applied for ESI-Orbitrap-LIT-MS experiments S-17

Table S7 Protein recoveries and total UV densities calculated for individual samples separated by SDS-PAGE..S-20

Table S10 Glycation sites in *A. thaliana* plants affected significantly by PEG osmotic stress ………………………………………………………………………………………...S-21

Table S11 Summary of protein homology modeling performed for the stress-specifically AGE-modified proteins peptides..S-23

Figure S1 Characterization of stress parameters in *A. thaliana* plants S-25

Figure S2 Relative contents of organic acids in *A. thaliana* leaf tissues S-26

Figure S3 Relative contents of carbohydrates in *A. thaliana* leaf tissues...........................S-27

Figure S4 The contents of free glyoxal and methylglyoxal in *A. thaliana* leaf tissues S-28

Figure S5 SDS-PAGE of individual *A. thaliana* leaf protein isolates S-29

Figure S6 SDS-PAGE of individual *A. thaliana* leaf protein tryptic digests......................S-30

Figure S7 Distribution of AGEs by clases in the proteins obtained from control *A. thalian*a plants S-31

Figure S8 Numbers of modified peptides representing specific AGE classes identified by MS/MS fragmentation patterns in *A. thaliana* drought-treated and control plants S-32

Figure S9 Functional annotation of the unique drought-specifically AGE-modified *A. thaliana* proteins...S-33

Figure S10 Functional annotation of the AGE-modified A. thaliana proteins demonstrating significantly ($p \leq 0.05$) different abundance of corresponding glycation sites S-34

Figure S11 Anti-dinitrophenylhydrazine Western blot analysis of protein carbonylation .S-35

Figure S12 The water potential and leaf relative water content of A. thaliana plants grown for three and seven days in presence and absence of PEG-induced drought S-36

Figure S13 Principal component analysis (PCA) of the primary metabolites and drought stress markers S-37

Calculations S1 Linear regression analysis S-38

Literature S-40

Protocols

Protocol S1

Quantification of lipid hydroperoxides (Griffiths *et al.* 2000 with changes).

Approximately 10 mg of frozen milled plant material were left for 5 min on ice, before 750 µL of ice-cold chloroform-methanol mixture (1:2, v/v) and 150 µL of 0.15 mol/L aq. acetic acid were added and the suspension was vortexed for 30 s. Then, chloroform and water (225 µL each) were added, the suspension was vortexed for 30 s and centrifuged at 3000 g for 5 min. The lower phase was collected, transferred to black polypropylene tubes and dried under nitrogen flow provided by a sample concentrator (Bibby Scientific Limited, Staffordshire, UK) for 30 – 60 min. The residue was reconstituted in 100 µL 0.01% butylated hydroxytoluene (BHT) in methanol and left on ice for 30 min before 900 µL of working FOX reagent (1.0 mmol/L xylenol orange and 2.5 mmol/L ammonium ferrous sulfate in 250 mmol/L H_2SO_4 – 0.01% BHT in methanol, 1 : 9, v/v) was added. After 30 min incubation on ice, absorption was measured at 650 nm against working FOX reagent. Hydroperoxide content was calculated as 13*S*-hydroperoxy-9*Z*, 11*E*-octadecanoic acid equivalents, ε = 6.0 x 10^4 $M^{-1}cm^{-1}$ (Gay *et al.* 1999).

Protocol S2

Approximately 100 mg of the plant material were extracted with 1 mL of ice-cold 0.4 mol/L perchloric acid. Samples were vortexed for 30 s and centrifuged (10 000 g, 10 min, 4 °C). The supernatant was neutralized with KOH, diluted four-fold with sodium phosphate buffer (0.1 mol, pH 5.6) and supplemented with ascorbate oxidase (8 units, 2 µL in 4 mmol/L sodium phosphate buffer pH 5.6, 10 min, RT). Afterwards, two aliquots (500 µL each) were transferred to new polypropylene tubes, with one of them treated with catalase (50 units in 2 µL in 4 mmol/L sodium phosphate buffer pH 5.6, 2 min, RT). Both aliquots were supplemented with an equal volume of the FOX reagent (0.2 mmol/L xylenol orange, 200 mmol/L sorbitol, 50 mmol/L H_2SO_4, and 0.5 mmol/L $(NH_4)_2 Fe(SO_4)_2$), and incubated for 30 min in the dark before measurement of the Fe(II)-xylenol orange complex absorption at 575 nm. The values obtained for the catalase-treated samples were subtracted from those of the catalase-free ones, to obtain the corrected optical densities. The calibration was performed externally by an H_2O_2 serial dilution series (1–10 µmol/L).

Protocol S3

Determination of malondialdehyde (MDA) contents (Velikova *et al* 2000 with changes).

In detail, approximately 25 mg of frozen grinded plant material were left on ice for 3 minutes, before addition of 300 µL 5% (w/v) trichloroacetic acid (TCA), vortexed for 30 s and centrifuged at 10000 g for 20 minutes at 4°C. 250 µL of supernatant were transferred in a new polypropylene tube, and 1000 µL of thiobarbituric acid (TBA) reagent (0.5 % w/v TBA in 20% TCA) were added. The mixture was incubated for 30 min in boiling water bath (95°C). Afterwards, the mixture was cooled on ice to stop the reaction, centrifuged at 1900 g for 10 minutes at 4°C and 1 ml of colored supernatant was used to measure the absorbance at 532 nm against the proper blank (250 µL 5% w/v TCA and 750 µL TBA reagent). The non-specific absorbance at 600 nm was subtracted from the absorbance acquired at 532 nm. The contents of MDA equivalents were calculated with $\varepsilon = 155\ mM^{-1}cm^{-1}$

Protocol S4

Determination of ascorbic and dehydroascorbic acid contents (Huang *et al.* 2005 with changes)

Approximately 50 mg of frozen plant material were left on ice for 5 min before 0.5 mL of ice-cold 2.5 mol/L $HClO_4$ were added. The suspensions were vortexed for 30 s and centrifuged for 10 min at 10000 g and 4°C. The supernatants were transferred in new polypropylene tubes, neutralized with saturated Na_2CO_3 solution and 10-fold diluted with 0.1 mol/L sodium phosphate buffer (pH 5.6). For determination of ascorbic acid, 500 µL of diluted extract were placed in a quartz cell and absorbance at 265 nm was recoded (Gemini EM microplate reader, Molecular Devices (Germany) GmbH, Biberach, Germany) before 1 u of ascorbate oxidase (i.e. 1 µL in 4 mmol/L sodium phosphate buffer) was added, and absorbance was recoded once more two minutes later. Total ascorbate was quantified after reduction of diluted extract with DTT (3 µL of 3 mol/L solution) for 1 min on ice at the same wavelength. Dehydroascorbic acid was calculated as the difference of the total ascorbate and ascorbic acid contents.

Protocol S5 Gene expression analysis

Total RNA was isolated from ~ 100 mg of frozen ground plant material using the NucleoSpin® RNA Plus kit (Macherey-Nagel GmbH & Co KG, Düren, Germany) according to manufacturer's instructions. The RNA concentrations and purity/integrity were determined spectrophotometrically at 260 and 280 nm (ND-1000, Nanodrop Technologies Inc, Wilmington, USA) and electrophoresis in 1.2% agarose gels, respectively. The cDNA synthesis was performed with 1.5 μg total RNA using the Maxima H Minus First Strand cDNA Synthesis kit (Thermo Fisher Scientific, Darmstadt, Germany) according the manufacturer's instructions. The polymerase chain reaction (PCR) was performed with 1 μL aliquots of sample cDNA using Plant direct 2x PCR Mastermix (Bio&SELL Nümberg, Germany). Primers were designed by OligoPerfect™ Designer with parameters set to amplify products of 100–120 bp with an optimal melting temperature of 53 °C and GC content between 40 and 60% (http://tools.invitrogen.com/content.cfm?pageid=9716). For primer sequences see Table S-1. The obtained PCR products were separated by electrophoresis on a 2% agarose gel.

For RT-qPCR, the cDNA samples were diluted 5-fold with sterile water, and amplification was performed in triplicates in Hard-Shell® 96-well plates (Bio-Rad, München, Germany) using 5x QPCR Mix EvaGreen® (No ROX) kit (Bio&SELL, Feucht bei Nümberg, Germany) according to manufacturer's instructions (see Protocol S-6 for details). The RT–qPCR data were collected and processed by Bio-Rad CFX Manager 2.1 software and normalized for the reference gene of actin (*ACT2, At5g09810*). The relative expression levels of genes in stressed plants were calculated using the $2^{-\Delta\Delta CT}$ method represented as relative fold changes in comparison to the expression levels of the control genes (Livak *et al.* 2001; Schmittgen *et al.* 2008). Actin gene *ACT2* was used as a reference gene.

Protocol S6 Determination of protein concentrations in a 96-microtiter plate format by the Bradford assay

The Bradford assay was performed in 96-well microtiter plates (MICROLON® 200, Greiner Bio-One GmbH, Frickenhausen, Germany). The protein extracts were serially diluted in polypropylene tubes with water using a 2-fold increment (from 1:4 to 1:128). For this, 10 µL of extract was mixed with 30 µL of water, and 20 µL were serially transferred in further tubes containing 20 µL of water. The method was calibrated with bovine serum albumin (BSA) dilution ranges (0.0625 – 1 mg/mL, n = 3) prepared in the same way. Calibration standards and serially diluted samples (5 µL) were pipetted in the wells of a 96-well plate and 250 µL of Bradford reagent were added in each well with a multi-channel pipette. After 15 min agitation in dark, absorption was determined at 595 nm with a microtiter plate reader (Tecan Group Ltd., Männedorf, Germany) and sample serial dilutions in water (up to 1:128 with a 2-fold increment). Thereby, quantification relied on dilution ranges prepared with bovine serum albumin (0.0625 – 1 mg/mL) in the same way.

Bradford reagent:

25.0 mg Coomassie Blue G-250

12.5 mL 96% ethanol

25.0 mL 85% H_3PO_4

212.5 mL H_2O

The mixture was incubated for 1 h at 60^{0}C and afterwards overnight at room temperature (RT). Ready solution was filtrated at least two times through a paper filter. Unused solution was stored at -20^{0}C.

Protocol S7 Sodium dodecyl sulfate-polyacrylamide gel electrophoresis (SDS-PAGE)

SDS-PAGE was done with a 12% resolving and a 6% stacking (gel T=12%, C=2.65%).[26] An aliquot (5 µg protein) of the sample was diluted with sample buffer (0.05% bromophenol blue, 62.5 mmol/L Tris-HCl, pH 6.8, 20% glycerol, 2% SDS, 5% β-mercaptoethanol) at least twofold and heated to 95°C for 5 min. Samples were diluted with sample buffer at least 1:3, heated to 95°C for 5 min, and an equivalent of 2 µg protein was loaded per lane. One lane per gel was loaded with non-digested HSA, another one with a molecular weight standard. Following separation (approximately 45 min at 200 V), gels were stained with Coomassie Brilliant Blue G 250.[27]

For Western blotting, 10 µg of protein extract were separated by SDS-PAGE using 0.75 mm-thick gel blocks (12% resolving and 6% stacking) containing 5% 2,2,2-trichloroethanol.

Protocol S8 Protein homology modeling

Protein homology modelling of all proteins listed in Table S-11 were automatically performed with YASARA (1). After search for templates in the protein database **(2**) for each protein, up to 100 models were created based on alternative sequence alignments including secondary structure predictions and comparisons with found appropriate X-ray protein structures. All these resulting models were evaluated by YASARA, and if appropriate a final model was created by merging best folded fragments from different models followed by energy minimization. The quality of all models was checked for native folding by energy calculations with PROSA II (3,4) and for stereo-chemical quality by PROCKECK (5). Since all sequences have a similarity to the best suited template for homology modelling higher than 30% all the models were of sufficient quality. For one sequence (Q9SB63) no model could be built due to missing sequence similarity to any protein with resolved 3D-structure. In Table S-11 the PDB-codes of all the best suited templates are listed together with their related sequence identities and similarities between target and template sequences. However, it has to be taken in to consideration that for the final model used for inspection of AEG positions several other template proteins contributed at least in some parts for the construction of the final models.

The models were manually inspected for the detection of the AEG modification sites by using the “molecular operating environment” program package MOE 2015.1001 (https://www.chemcomp.com/).

Tables

Table S1 Primer sequences for target and reference genes used in RT-qPCR experiments

Gene symbol/protein product	Arabidopsis genome initiative identifier	qRT-PCR amplicon size (bp)	Orienta-tion	5’–3’ sequence (20 bp)	GC (%)	T_m (°C)
GLX1/Glyoxalase I	*At1G08110*	111	Forward	CGAGGATACTACAACAGCTC	50	57.3
			Reverse	TCAGGATCACTCTCTGTACC	50	57.3
GLX2/Glyoxalase II	*At2G43430*	121	Forward	ATGAGGTTCGGATACTTGAC	45	55.3
			Reverse	GAAAGGGTACCACAGGATAA	45	55.3
APX1/Ascorbate peroxidase, cytosolic	*At1G07890*	126	Forward	CAAACCCTCTAATCTTCGAC	45	55.3
			Reverse	GTATTTCTCGACCAAAGGAC	45	55.3
GRcyt/Glutathione reductase, cytosolic	*At3G24170*	124	Forward	GAAGTGGAGGTGAGACAAAT	45	55.3
			Reverse	CAGATGTAATAGCCAGCTCA	45	55.3
NCED3/ 9-cis-epoxycarotenoid dioxygenase 3	*At3g14440*	120	Forward	CACGACGAGAAGACATGGAA	50	57.3
			Reverse	TCCGATGAATGTACCGTGAA	45	55.3
Reference gene primers						
ACT2/Actin 2	*At5g09810*	130	Forward	GCCAGAGAGAAAATACAGTG	45	55.3
			Reverse	ACCTGACTCATCGTACTCAC	50	57.3

Table S2 Gas chromatographic (GC) separation conditions and electron ionization-quadrupole-mass spectrometry (EI-Q-MS) settings for analysis of *A. thaliana* metabolites

**Parameters**	**Setting**	
	**GC settings**	
	**Carbohydrate analysis**	**Carbonyl analysis**
Separation column	HP-5 capillary column (30 m × 0.25 mm ID, 0.25 µm film thickness, Thermo Fisher Scientific, Bremen, Germany)	HP 19091j-433 column (Agilent Technologies, USA)
Carrier gas / carrier gas flow rate	Helium / 1 mL/min	Helium / 1 mL/min
Injector operation mode	Splitless mode (90 s splitless time)	Splitless mode (2 min splitless time)
Injector temperature	250°C	250°C
Temperature program	1 min at 40°C<br>ramp 15°C/min to 70°C<br>1 min at 70°C<br>ramp 6°C/min to 320°C<br>10 min at 320°C	2 min at 50°C<br>ramp 10°C/min to 325°C<br>15 min at 325°C
**Parameters**	**MS settings**	
Ionization mode	Electron ionization (EI)	Electron ionization (EI)
Electron energy	70 eV	70 eV
Operation mode	scanning at 0.34 sec $scan^{-1}$	scanning at 1 sec $scan^{-1}$
m/z range	50 - 550	50-800

Table S3 Parameters of the HILIC separation method

Parameter	Setting
Method parameters	
Injection volume	290 µL
Injection mode	Microliter pick-up
Eluents	A: 90% (v/v) CH_3CN in H_2O, 20 mM $(NH_4)HCOO$, pH 3.2<br>B: 50% (v/v) CH_3CN in H_2O, 40 mM $(NH_4)HCOO$, pH 3.2<br>C: 50% (v/v) CH_3CN in H_2O
Elution flow rate	0.1 mL/min
Column temperature	RT
Elution regimen	Isocratic 0% B – 20 min<br>Linear gradient – 0 to 50% B in 10 min<br>Linear gradient – 50 to 100% B in 50 min<br>Isocratic 100% B – for 5 min<br>Isocratic 100% C – for 10 min
Re-equilibration	Isocratic 0% B – for 35 minutes

Table S4 Reconstitution of HILIC fractions for nanoUPLC-ESI-Orbitrap-LIT-MS/MS experiments

Fraction	Sample reconstitution	Sample dilution		Further dilution[b]
	60% CH_3CN (v/v) in 0.1% formic acid (v/v) (µl)	Added 0.1% (v/v) aq. formic acid (µl)	Final volume (µl)	
1	25	475 (as 75, 200, 200)[a]	500	-
2	37.5	712.5 (as 112.5, 300, 300)[a]	750	-
3	37.5	712.5 (as 112.5, 300, 300)[a]	810	3-fold
4	37.5	712.5 (as 112.5, 300, 300)[a]	795	3-fold
5	25	475 (as 75, 200, 200)[a]	500	-

[a]after the addition of each portion, the samples were vortexed 30 s and centrifuged 1 min at 10000 rpm; [b]performed with 3% (v/v) acetonitrile in 0.1% (v/v) aq. formic acid

Table S5 Parameters of the nanoUPLC separation method

Parameter	Setting
Method parameters (AGE identification)	
Injection volume	10 µL
Injection mode	Full loop injection
Trapping flow rate	5 µL/min
Trapping duration	5 min
Eluents	A: 0.1% (v/v) aq. formic acid; B: 0.1% (v/v) formic acid in acetonitrile
Elution flow rate	0.4 µL/min
Column temperature	30°C
Elution regimen	Isocratic 3% eluent B – 5 min<br>Linear gradient - from 3 to 50% eluent B in 45 min<br>Linear gradient – from 50 to 85% eluent B in 2 min
Re-equilibration	Isocratic 3% eluent B – 10 min
Method parameters (Protein quantitation)	
Trapping coloumn	C_8 PepMap^{TM} 100 µ-precolumn, particle size 5 µm, pore size 100 Å (Thermo Fisher Scientific, Bremen, Germany)
Seperation coloumn	Acclaim PepMap^{TM} 100, ID 75 µm, length 150 mm, particle size 3 µm, pore size 100 Å (Thermo Fisher Scientific)
Injection volume	30 µL
Injection mode	Micro liter pick-up
Trapping flow rate	20 µL/min
Trapping duration	15 min
Eluents	A: 5% (v/v) aq. acetonitrile with 0.1% (v/v) formic acid;<br>B: 80% (v/v) aq. acetonitrile with 0.08% (v/v) formic acid
Elution flow rate	0.3 µL/min
Column temperature	40°C
Elution regimen	Linear gradient - from 0 to 60% eluent B in 45 min<br>Linear gradient – from 60 to 100% eluent B in 2 min
Re-equilibration	Isocratic 0% eluent B – 15 min

Table S6 Instrument settings applied for ESI-Orbitrap-LIT-MS experiments

Parameter	Setting
MS conditions (AGE Identification)	
Ionization mode	Positive
ESI emitter	outer diameter 360/20 µm, 10 µm internal diameter (New Objective, Berlin, Germany)
Resolution	60000
Ion spray voltage (IS)	1500 V
Aux gas flow rate	1 arb
Capillary temperature	200 °C
Tube lens voltage	120 V
Mass to charge ratio (*m/z*) range	400 – 2000
MS conditions (Protein quantitation)	
Ionization mode	Positive
ESI emitter	Stainless steel emitter (Thermo Fisher Scientific)
Resolution	60000
Ion spray voltage (IS)	1900 V
Aux gas flow rate	1 arb
Capillary temperature	200 °C
Tube lens voltage	115 V
Mass to charge ratio (*m/z*) range	400 – 2000
MS/MS conditions	
Fragmentation	Collision activated dissociation
Isolation width	2 Da

Charge state rejected	1+
Normalized collision energy	35%
Activation frequency	0.25
Activation time	30 ms
Parent mass width	± 0.5 Da
Reject mass width	± 5 ppm
Dynamic exclusion repeat count	1
Dynamic exclusion repeat duration	30 s
Dynamic exclusion duration	3600 s
Dynamic exclusion mass width	± 5 ppm
Database search settings	
Analysis program	SEQUEST
Protease	Trypsin
Missed cleavage sites	3
Modification	Mass increment (Da) / amino acids
Carbamidomethyl	+57.021/ C
Oxidation	+15.995 / C, M and W
Dioxidation	+31.990 / C, M and W
Trioxidation	+47.985 / C and W
Tryp->kynurenin	+3.995 / W
Tryp->oxolactone	+13.9792 / W
Tryp->hydroxykynurenin	+19.990 / W
Argpyrimidine	+80.026 / R
Carboxymethyl arginine/lysine	+58.01 / R, K
Glarg	+39.995 / R
MGH	+54.011/ R
Tetrahydropyrimidine	+144.042 / R
Pyrraline	+108.021 / K

GLAP	+109.029 / K
Carboxyethyl arginine/lysine	+72.0211/ R, K
Peptide search filters	2.20 for doubly, and 3.75 for quadruply and quantiply charged peptides, respectively
Protein search filters	1 or 3 peptides

Table S7 Protein recoveries and total UV (595 nm) densities calculated for individual samples separated by SDS-PAGE

**Sample**	**Weight (mg)**	**Concentration (mg/mL)**	**Recovery (mg/g fresh weight)**	**Intensity**
Control-1	510	3.7	1.5	2324410
Control-2	480	2.5	1.0	3308195
Control-3	476	2.5	1.1	2628615
Stress-1	453	1.5	0.7	1855095
Stress-2	472	3.5	1.5	2389365
Stress-3	471	2.1	0.9	1920105

Table S10 Glycation sites affected significantly ($p < 0.05$) in *A. thaliana* plants grown for three days on 0.8% agar infused with 172.27 g/L PEG 8000 (Ψ_w = -0.4 MPa) in comparison to those grown in PEG-free medium

Nr	Peptide Sequence	*m/z*	*z*	XCorr	t_R	Control			Stress			Change			Protein annotation[a]	
						Average	SD	RSD	Average	SD	RSD	Value (fold)	*p*-value	Direction	Accession number	Protein Name
1	YIYS[CEA]LDEWSK	774.379	2	2.44	21.3	857097	117197	14	489477	78854	16	1.8	0.011	↓	Q9ZPI5	Peroxisomal fatty acid beta-oxidation multifunctional protein MFP2[1]
2	G[CML]EEAWTDDQLFFTWK	1053.958	2	2.31	26.3	48006	17157	36	101849	18553	18	2.1	0.040	↑	F4I1L3	Acetyl-CoA carboxylase 2[1]
3	ILNIE[Glarg]K	463.277	2	2.28	22.5	1467717	346189	24	5571073	152332	3	3.8	0.001	↑	Q9SS38	DNA gyrase subunit B, chloroplastic[2]
4	EDDSKRGMISKIEAGGD[Argpyr]	1030.479	2	2.34	22.7	591455	83104	14	376388	93569	25	1.6	0.040	↓	Q9S7R7	BTB/POZ domain-containing protein At3g09030[3]
5	MACRAKELVSLIL Y[GLAP]	986.019	2	2.34	22.9	40100	26061	65	1101593	449916	41	27.5	0.050	↑	Q9XIA2	F-box protein At1g49360[3]
6	ELIE[MG-H]HCGG V[CMA]	719.348	2	2.23	24.7	152564	47573	31	60860	27750	46	2.5	0.040	↓	Q9FNN5	NADH dehydrogenase [ubiquinone] flavoprotein 1, mitochondrial[4]
7	MDKGSLG[MG-H]YALSRLVNM[MG-H]	1095.049	2	2.44	30.8	694862	138954	20	456775	48200	11	1.5	0.049	↓	Q94F87	DNA (cytosine-5)-methyltransferase CMT2[5]

8	NNNDLSAVSMNLLTPSVVA[CMA]	1095.055	2	2.61	31.1	694862	138954	20	456697	48286	11	1.5	0.049	↓	Q8S9L0	Squamosa promoter-binding-like protein 10[6]
9	ALREAMCFMMDPQS G[CML]K	1016.945	2	2.23	31.4	836550	158658	19	1186917	144853	12	1.4	0.048	↑	Q8L778	Cellulose synthase A catalytic subunit 5 [UDP-forming][7]
10	FLCDLNLTPEELVSTSTQ[CMA]	1128.547	2	2.24	32.8	13830799	2132732	15	7936464	1953605	25	1.8	0.024	↓	Q9SR66	DEMETER-like protein 2[8]
11	ELPDGLRFIYSLKNLIMG[GLAP]	1158.628	2	2.31	33.3	973721	320205	33	467005	47964	10	2.1	0.053	↓	Q9C646	Probable disease resistance protein RXW24L[9]
12	G[TH-Pyr]KLFW[GLAP] CEELIDK	1017.514	2	2.28	33.9	143109	9476	7	73010	14043	19	2.0	0.028	↓	Q9LUB7	Protein OBERON 2[10]

[a]The peptides are listed in order of protein functional annotation: [1]lipid metabolism; [2]remove DNA supercoils; [3]protein ubiquitination and degradation; [4]energy metabolism; [5]DNA methylation; [6]development; [7]cell wall formation; [8]regulation of transcription; [9]stress; [10]cell organization

Table S11 Summary of protein homology modeling performed for the stress-specifically AGE-modified proteins peptides.

Nr.[a]	Accession number[b]	Protein name	PDB code[c]	Sequence cover	Identity (%)	Similarity (%)
1[d]	Q5IBC5	Separase	5FBY	1585-2176	34.2	50.0
2[d]	Q3EDA9	Putative pentatricopeptide repeat-containing protein At1g16830	4M57	1-608	17.0	36.8
3	Q9SB63	Protein MODIFIER OF SNC1 1	-	-	-	-
4[d]	Q9M3B6	Plastidial pyruvate kinase 4, chloroplastic	4IQP7	166-710	34.3	51.7
5[d]	Q9SIN9	Phospholipase A1-Ialpha2, chloroplastic	2YIJ	80-484	35.0	54.4
6[d]	Q9SVL0	Zinc-finger homeodomain protein 7	1WH7	38-249	61.4	73.7
7[d]	Q9LHJ9	Probable protein phosphatase 2C 38	2PNQ	1-377	29.5	49.8
8[e]	Q9XGZ0	NADP-dependent malic enzyme 3	1PJ3	38-588	51.9	70.1
9[d]	Q9LH74	Mechanosensitive ion channel protein 5	5AJI	212-878	19.6	38.4
10[d]	Q8H166	Thiol protease aleurain	1CJL	42-358	41.8	58.8
11[f]	Q39189	DEAD-box ATP-dependent RNA helicase 7	4KBF	100-471	45.5	60.8
12	Q9FFK8	NF-X1-type zinc finger protein NFXL2	4XBM	40-759	26.7	39.8
13[d]	Q3EDF8	Pentatricopeptide repeat-containing protein At1g09900	4M57	1-598	24.4	46.8
14	Q9SK74	Zinc finger CCCH domain-containing protein 21	4A9A	202-332	30.2	46.5
15[g]	P23321	Oxygen-evolving enhancer protein 1-1, chloroplastic	3JCU	81-332	84.5	92.1
16	Q9LYN8	Leucine-rich repeat receptor protein kinase EMS1	4OH4	852-1192	55.8	72.5
17[d]	O03042	Ribulose bisphosphate carboxylase large chain	2V67	1-479	88.0	93.8
18[h]	Q96524	Cryptochrome-2	1U3C	1-502	60.2	78.6
19[d]	Q9ZT82	Callose synthase 12	4G72	4-913	14.7	29.1
20[d]	Q9M2Q4	RNA cytidine acetyltransferase 2	2ZPA	1-963	24.8	41.4
21[d]	P10795	Ribulose bisphosphate carboxylase small chain 1A, chloroplastic	1IR1	46-180	76.7	88.3
22[h]	Q96291	2-Cys peroxiredoxin BAS1, chloroplastic	5JCG	62-266	56.8	74.5

23	Q9SFX2	U-box domain-containing protein 43	2GL7	43-719	21.4	39.2
24[f]	Q3E8E5	Putative myrosinase 3	1MYR	11-439	60.2	71.4
25[d]	Q7XJK5	Agamous-like MADS-box protein AGL90	3P57	1-320	29.1	53.5
26[d]	Q9LER0	Pentatricopeptide repeat-containing protein At5g14770, mitochondrial	4PJQ	265-530	41.3	61.2
27[h]	Q0WRJ7	Peptidyl-prolyl cis-trans isomerase FKBP20-2, chloroplastic	1Q6H	9-235	23.0	44.5
28[d]	Q9C522	ATP-citrate synthase beta chain protein 1	3PFF	1-343	51.8	70.6
29	O22785	Pre-mRNA-processing factor 19 homolog 2	4LG8	1-519	46.2	65.5
30	Q9STT6	ABC transporter A family member 6	4YER	599-925	39.5	57.5
31[d]	Q84VG6	Pentatricopeptide repeat-containing protein At2g17525, mitochondrial	4M59	1-626	21.5	43.7

[a]The peptides are listed as in Table 1; [b]Uniprot accession numbers are given; [c]protein structures taken from the protein data bank (2); [d]modified residue is located on the protein surface; [e]modified residue is located in the N-terminal domain; [f]modified residue is located in the substrate-binding site; [g]modified residue is located in the photosystem stabilizing domain; [h]modified residue is located in the catalytic domain

Figures

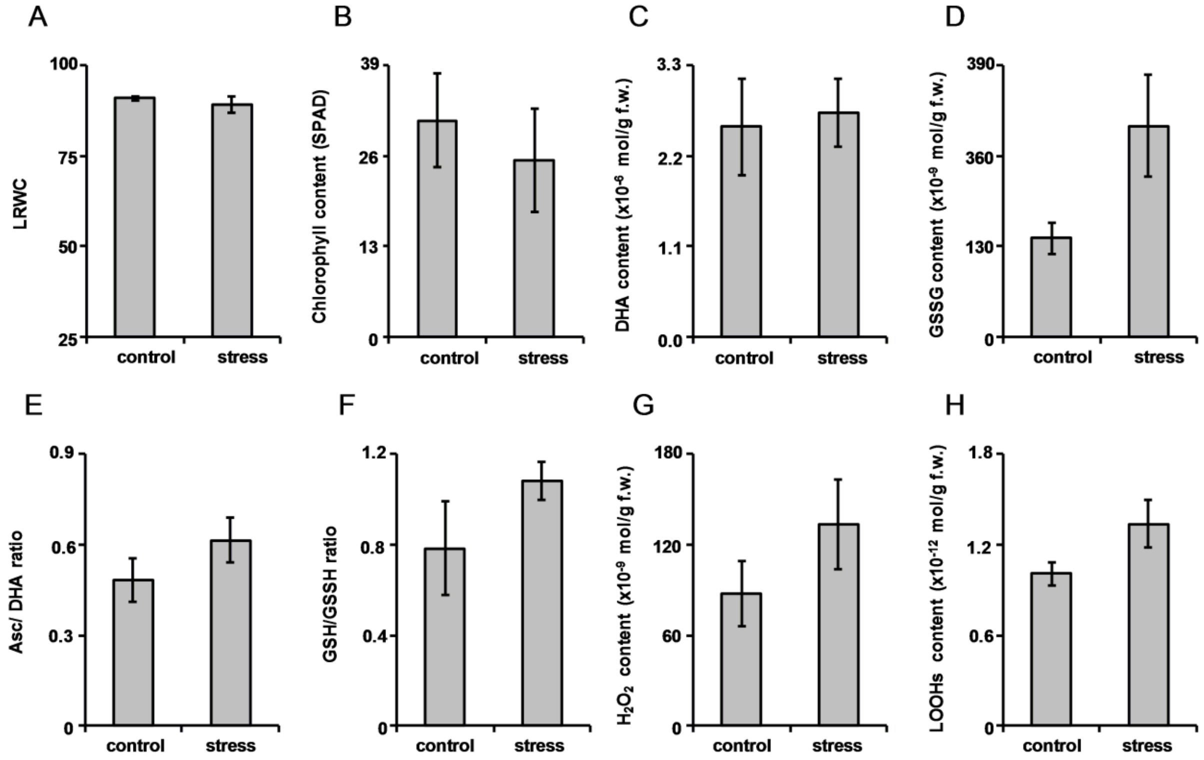

Figure S1 Characterization of the plant stress developed three days after the transfer of *A. thaliana* plants on agar medium saturated with PEG-free (control) and PEG 8000 solutions with ψ = -0.4 MPa (172.27 g/L) by the leaf relative water content (A), chlorophyll content (B), the tissue contents of dehydroascorbic acid (C), oxidized glutathione GSSG (D), Asc/DHA and GSH/GSSG ratios (E and F, respectively), as well as hydrogen peroxide (G) and lipid hydroperoxide (H) contents.

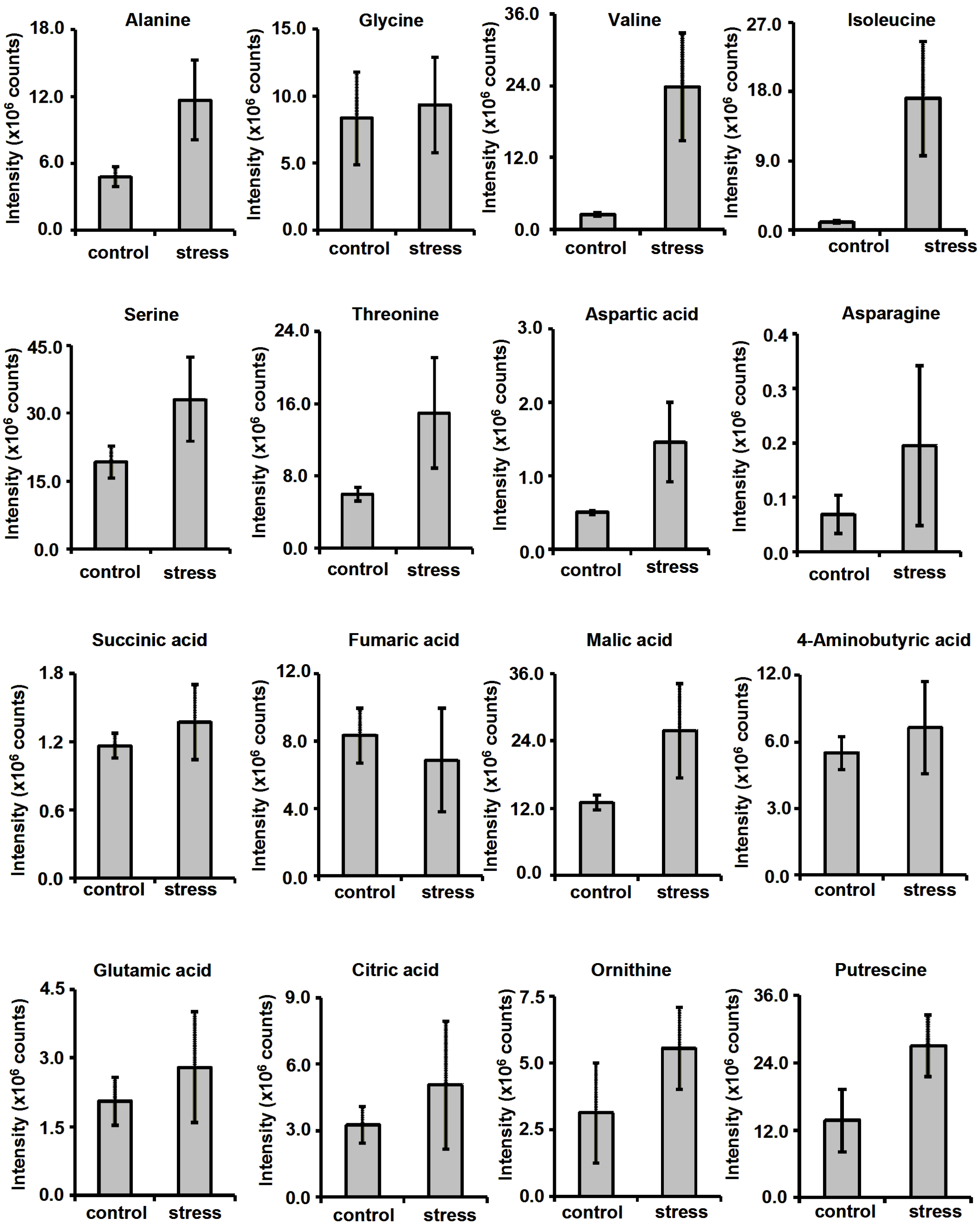

Figure S2 Relative contents of selected organic acids in the leaves of *A. thaliana* three days after the transfer to the 0.8% agar infused with the half-strength Murashige and Skoog medium in 6 mmol/L MES buffer (pH 5.7) in absence and presence of 172.27 g/L PEG 8000 (Ψ_w = -0.4 MPa)

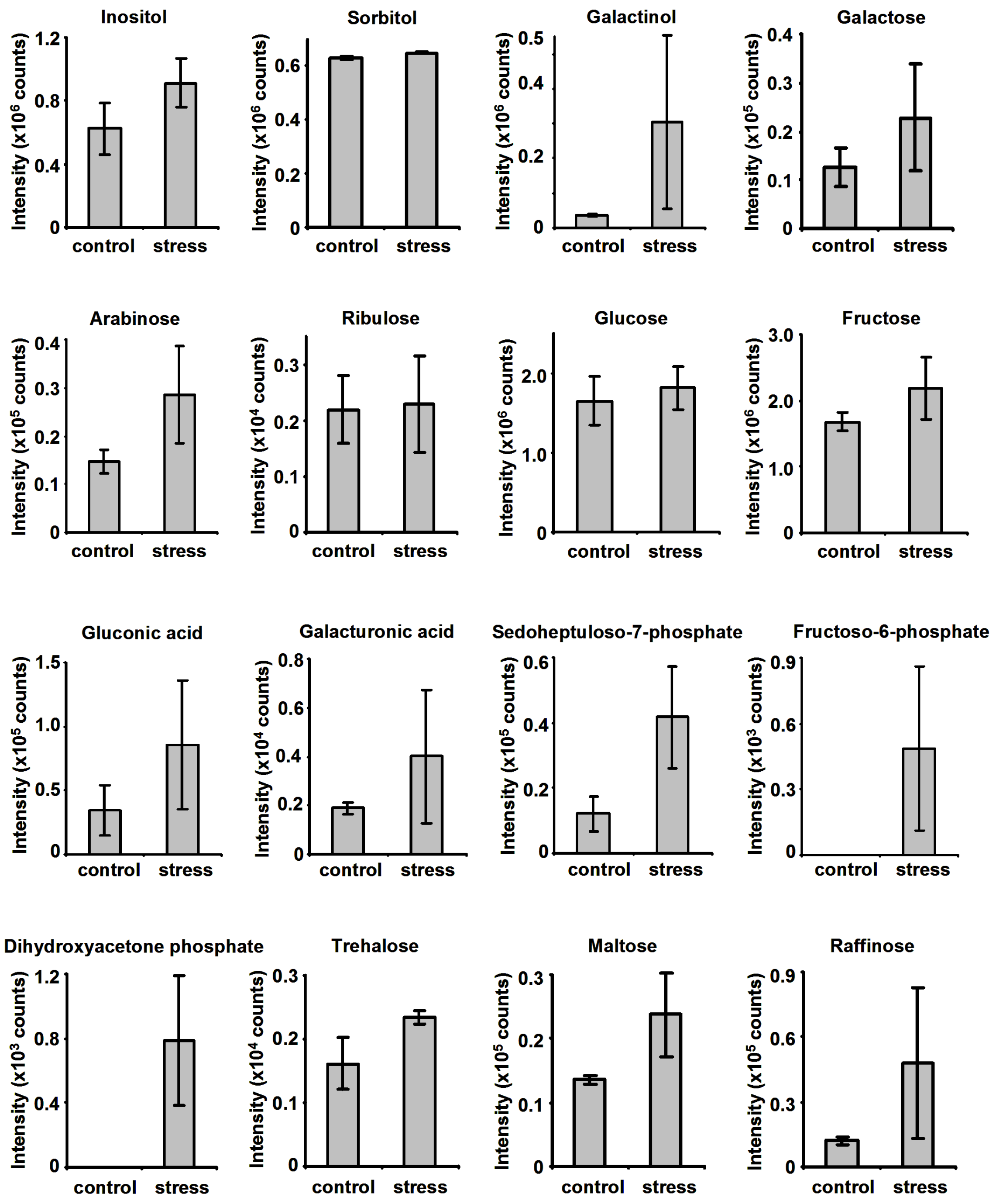

Figure S3 Relative contents of selected carbohydrates and polyols in the leaves of *A. thaliana* three days after the transfer to the 0.8% agar infused with the half-strength Murashige and Skoog medium in 6 mmol/L MES buffer (pH 5.7) in absence and presence of 172.27 g/L PEG 8000 (Ψ_w = -0.4 MPa)

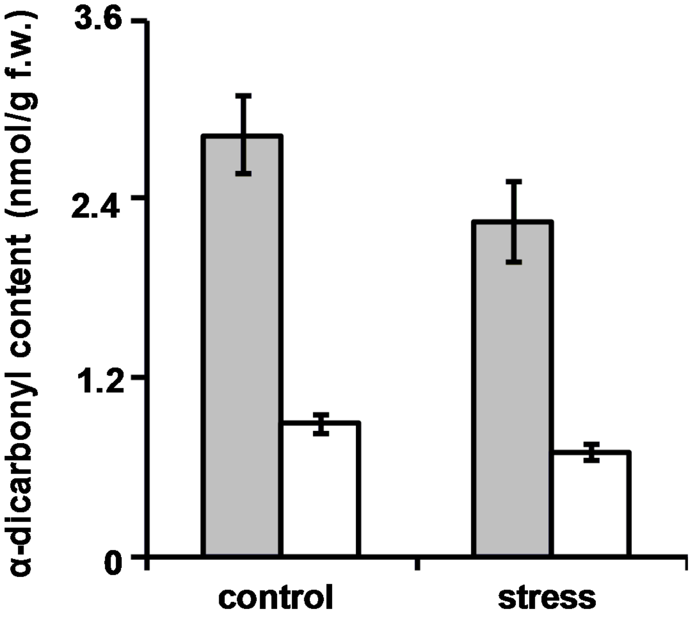

Figure S4 The contents of free glyoxal (grey) and methylglyoxal (white) in the leaves of *A. thaliana* observed three days after the transfer to the 0.8% agar infused with the half-strength Murashige and Skoog medium in 6 mmol/L MES buffer (pH 5.7) in absence and presence of 172.27 g/L PEG 8000 (Ψ_w = -0.4 MPa)

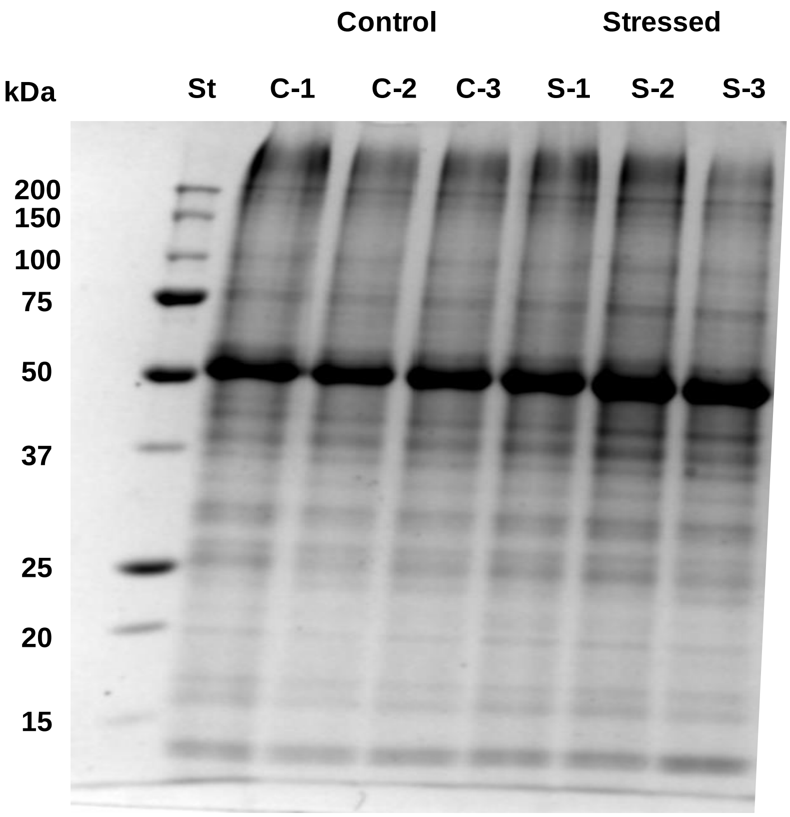

Figure S5 SDS-PAGE electropherogram of individual protein samples (5 µg) isolated from *A. thaliana* leaves

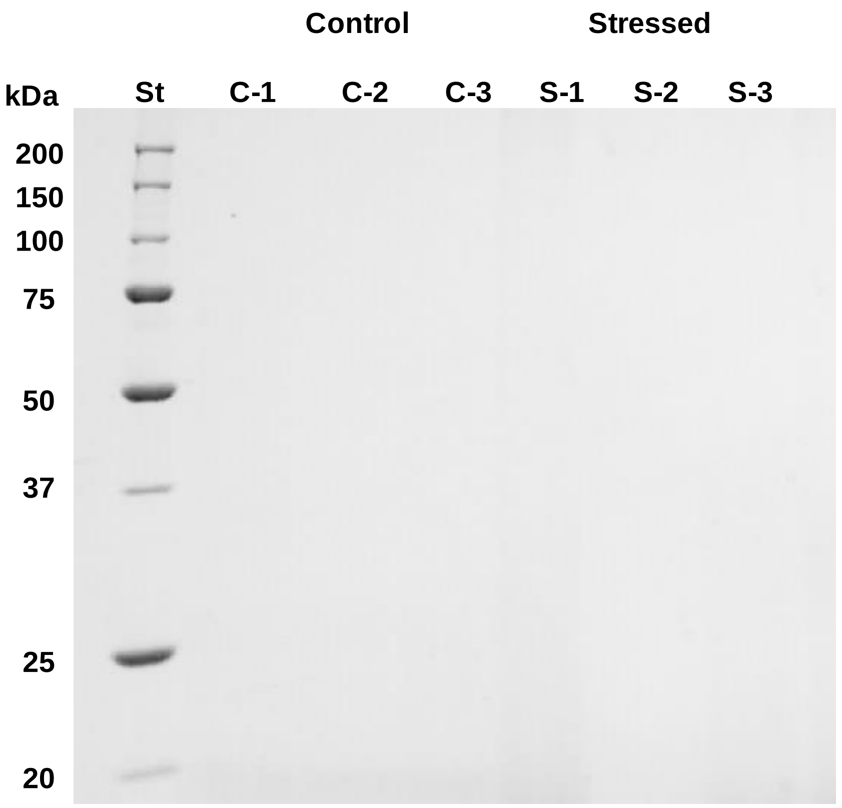

Figure S6 SDS-PAGE electropherogram of tryptic protein digests (5 µg) obtained from *A. thaliana* individual protein extracts

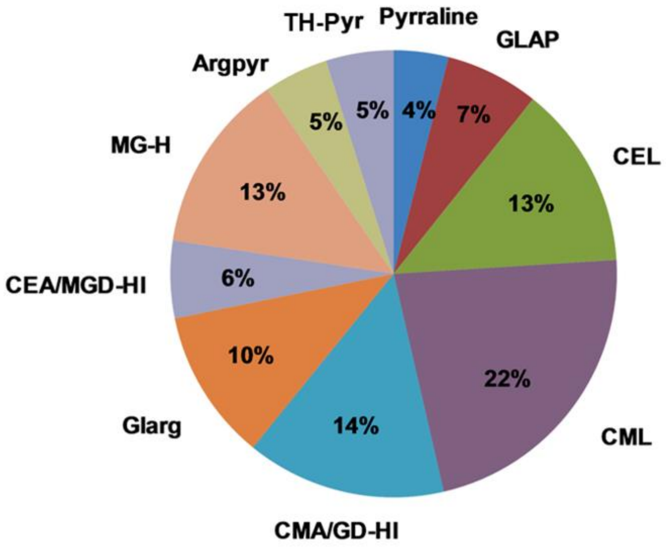

Figure S7 Distribution of AGE-modified proteins obtained from control *A. thaliana* plants by individual AGE classes, CML, N^{ε}-(carboxymethyl)lysine; CEL, N^{ε}-(carboxyethyl)lysine; pyrraline, ε-(2'-formyl-5'-hydroxymethyl-pyrrolyl)-L-norleucine; GLAP, glyceraldehyde-derived pyridinium compound; CMA, N^{δ}-(carboxylmethyl)arginine; GD-HI, glyoxal-derived hydroimidazolinone; M-GH, N^{δ}-(5-methyl-4-oxo-5-hydroimidazolinone-2-yl)-L-ornithine; Glarg, 1-(4-amino-4-carboxybutyl)2-imino-5-oxo-imidazolidine; CEA, N^{δ}-(carboxyethyl)arginine; MGD-HI, methylglyoxal-derived hydroimidazolinone; Argpyr, N^{δ}-(5-hydroxy-4,6-dimethylpyrimidine-2-yl)-*L*-ornithine; TH-Pyr, N^{δ}-(4-carboxy-4,6-dimethyl-5,6-dihydroxy-1,4,5,6- tetrahydropyrimidine-2-yl)-L-ornithine

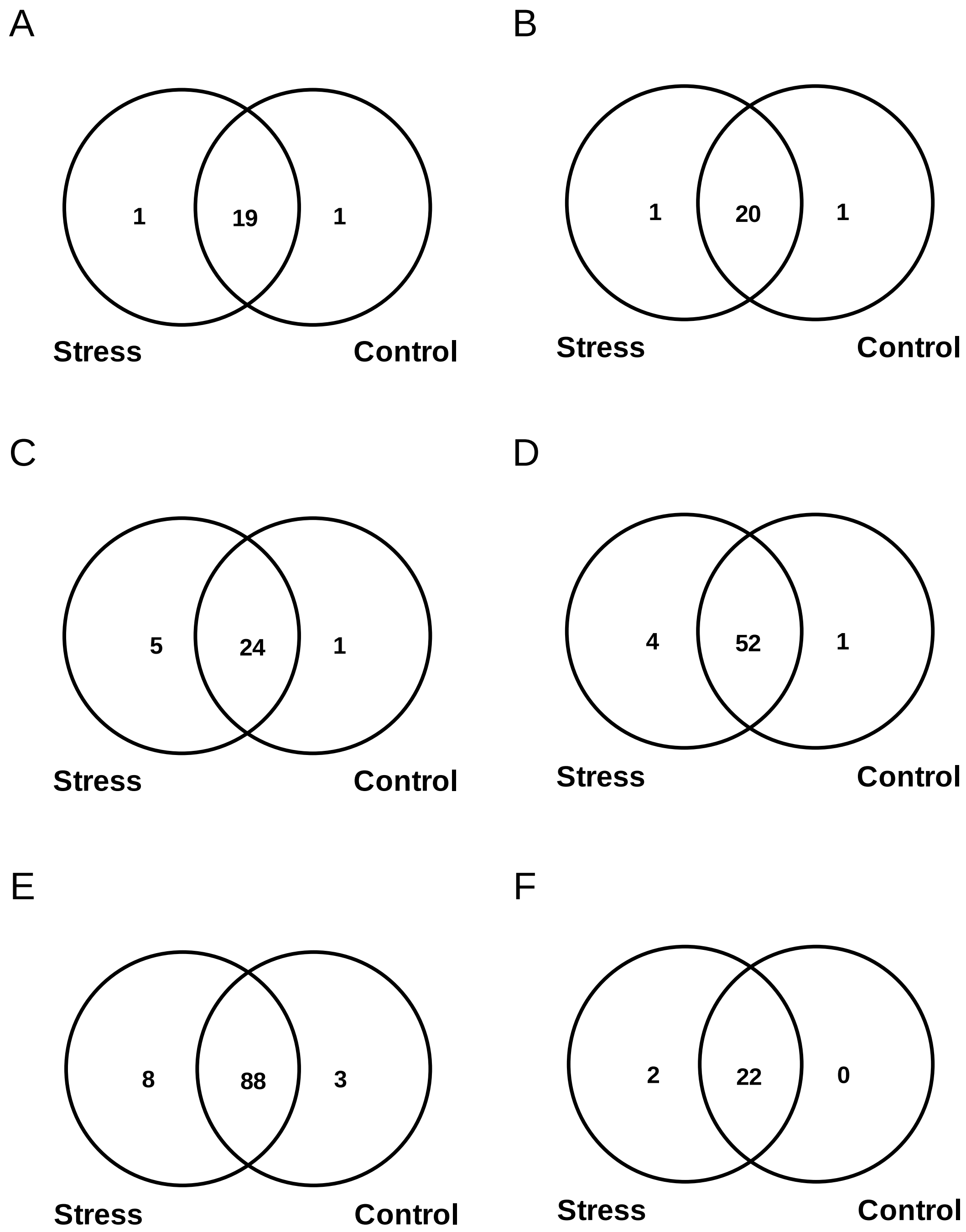

Figure S8 Numbers of modified peptides representing specific AGE classes identified by MS/MS fragmentation patterns in *A. thaliana* drought-treated and control plants: Argpyr (A), TH-Pyr (B), GLAP (C), MG-H (D), CML (E), and CEA/MGD-HI (F)

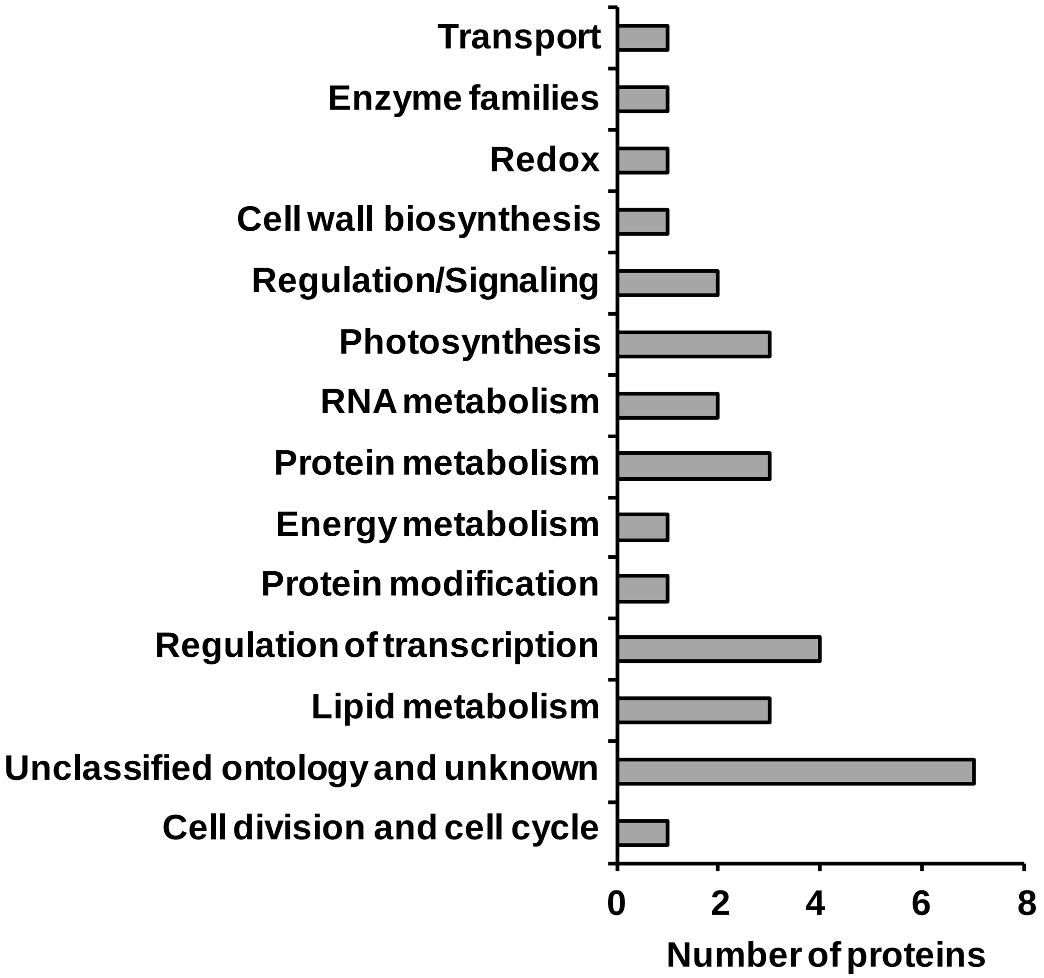

Figure S9 Functional annotation of the unique drought-specifically AGE-modified *A. thaliana* proteins

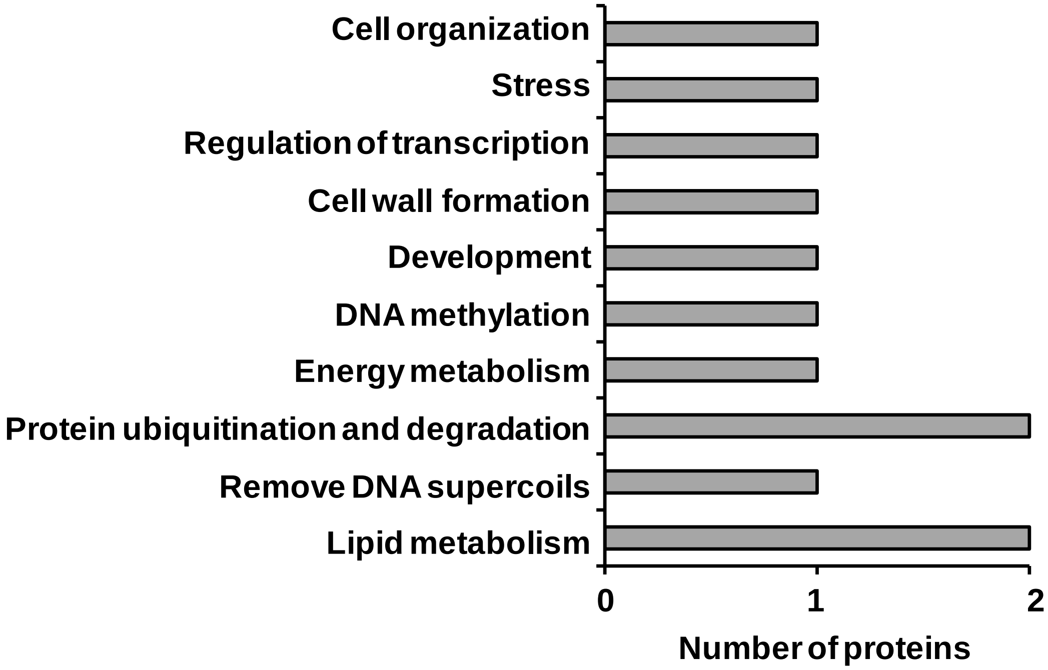

Figure S10 Functional annotation of the AGE-modified *A. thaliana* proteins demonstrating significantly ($p \leq 0.05$) different abundance of corresponding glycation sites

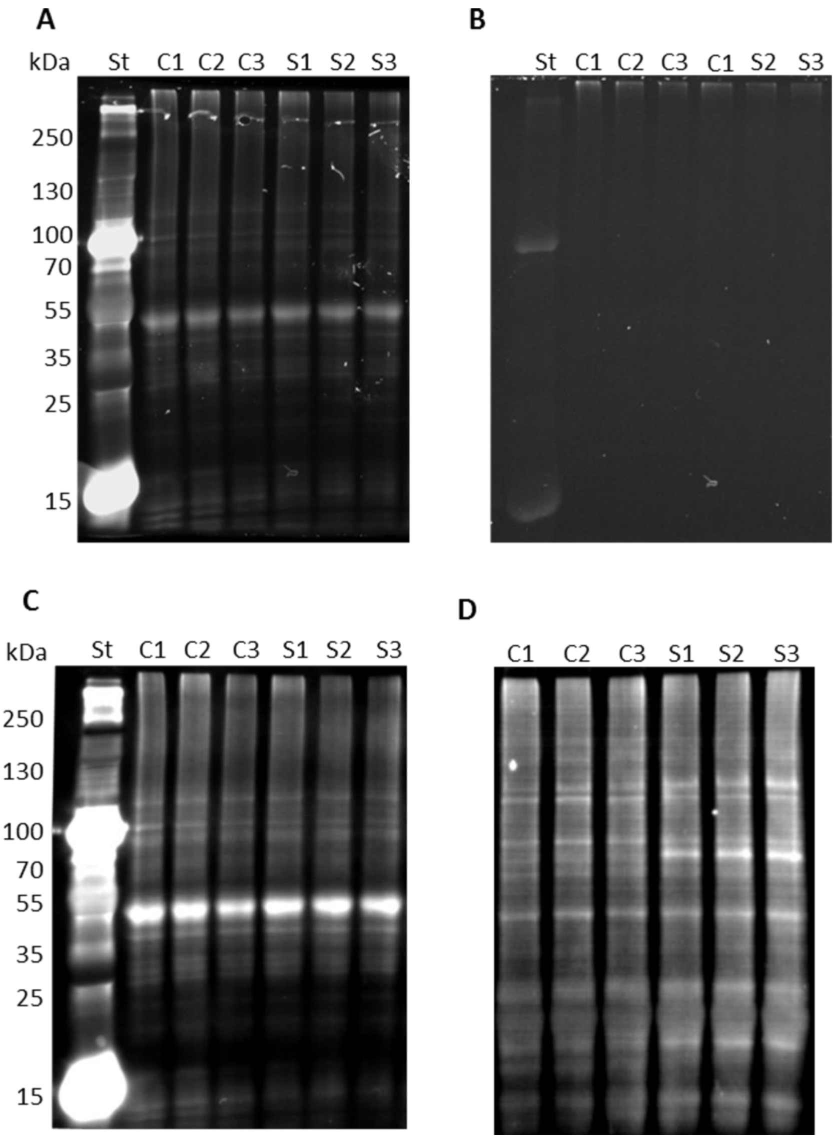

Figure S11 Anti-dinitrophenylhydrazine (DNP) Western blot analysis of protein carbonylation: the TCE-fluorescence image of the polyacrylamide gel obtained before (A) and after (B) protein transfer to membrane , the TCE-fluorescence image of the PVDF membrane obtained after protein transfer (C), the protein band with the highest TCE-fluorescence at A and C presents the most abundant leaf protein RuBisCO large chain; the membrane secondary antibody fluorescence image indicating carbonylated proteins (D)

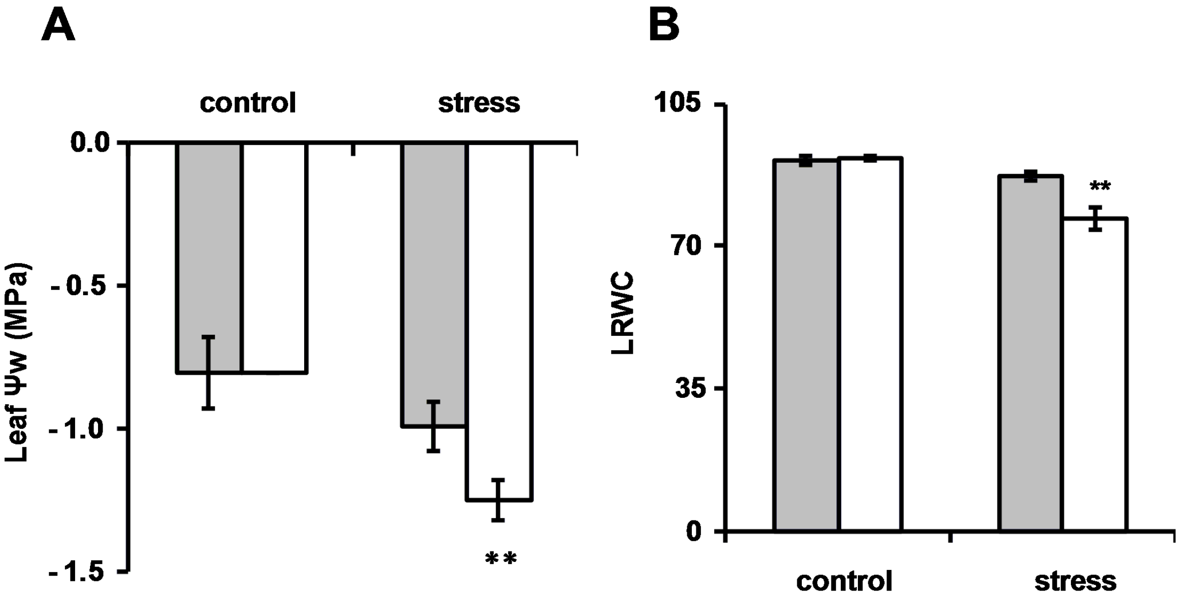

Figure S12 The water potential (ψ_w, A) and leaf relative water content (LRWC, B) *A.* of *thaliana* plants grown for three (grey) and seven days (white) on a 0.8% agar medium infused with half-strength Murashige and Skoog medium in 6 mmol/L MES buffer (pH 5.7) in presence of 172.27 g/L PEG 8000 (overlay ψ_w = -0.4 MPa). The ψ_w was determined by the gravimetric method of Rayle and co-workers (Rayle *et al.* 1982). ** represents statistical significance on the confidence level p<0.01

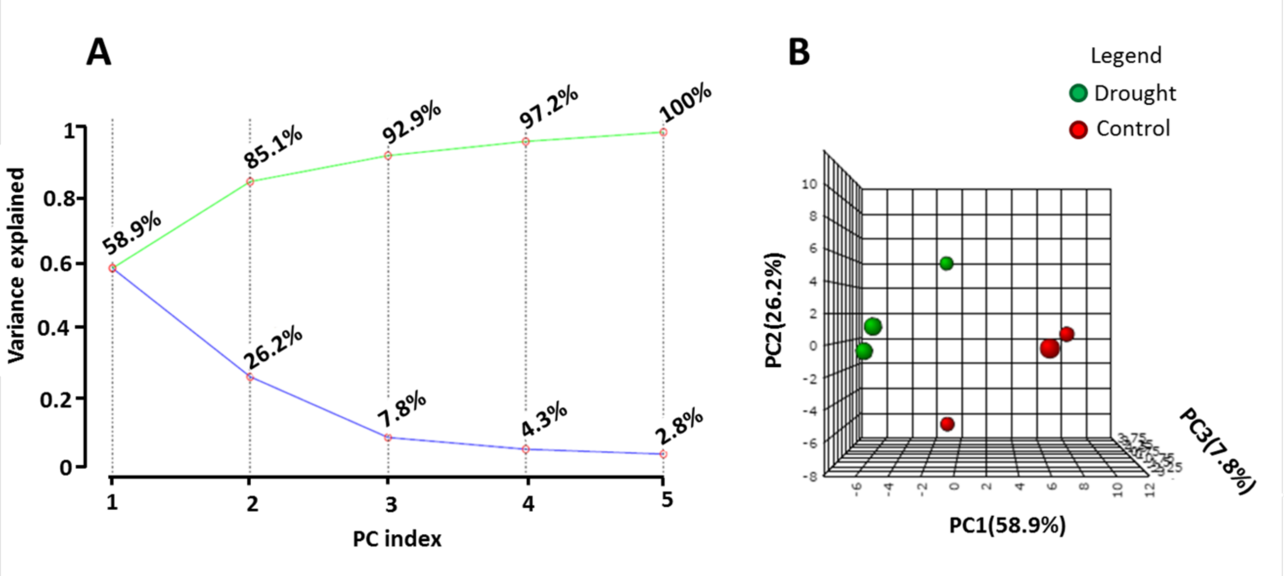

Figure S13 Principal component analysis (PCA) of the 59 primary metabolites and drought stress markers annotated in the leaf tissue methanolic extracts obtained from *A. thaliana* plants grown for three days on 0.8% agar infused with the half-strength Murashige and Skoog medium in 6 mmol/L MES buffer (pH 5.7) in absence and presence of 172.27 g/L PEG 8000 (Ψ_w = -0.4 MPa). The PCA analysis was performed by MetaboAnalyst 3.0 on-line tool (http://www.metaboanalyst.ca/) (6). A – Screen plot. The variance explained by individual principal component (PC) is shown with blue line, whereas the green line presents the summarized variance explained. B – 3D Score plot of the first three components, covering 58.9, 26.2 and 7.8% of the total variance.

Calculations

Calculations S1 Linear regression analysis

Linear regression analysis was performed on the base of MATLab 2016A (http://www.mathworks.com/) (7)

Linear regression models for MDA and GSH versus sugar relative metabolites. 31 sugar metabolites quantified on the base of GC-MS data were analyzed with PCA prior to the linear regression test to reduce the dimensionality, and only two first principle components were taking into account.

1) MDA

Linear regression model: MDA ~ 1 + PC1

Estimated Coefficients:

	Estimate	SE	tStat	pValue
(Intercept)	24.227	1.7608	13.759	0.000162
x1	19.889	4.3129	4.6116	0.009944

Number of observations: 6, Error degrees of freedom: 4

Root Mean Squared Error: 4.31

R-squared: 0.842, Adjusted R-Squared 0.802

F-statistic vs. constant model: 21.3, p-value = 0.00994

2) GSH

Linear regression model: GSH ~ 1 + PC2

Estimated Coefficients:

	Estimate	SE	tStat	pValue
(Intercept)	593.58	43.934	13.511	0.000174
x1	-505.16	107.61	-4.6941	0.009349

Number of observations: 6, Error degrees of freedom: 4

Root Mean Squared Error: 108

R-squared: 0.846, Adjusted R-Squared 0.808

F-statistic vs. constant model: 22, p-value = 0.00935

Literature

1) Krieger, E. *et al.* Improving physical realism, stereochemistry, and side-chain accuracy in homology modeling: Four approaches that performed well in CASP8. *Proteins* **9**, 114-122 (2009).

2) Berman, H. M. *et al.* The Protein Data Bank. *Nucleic Acids Res* **28**, 235-242 (2000). (http://www.rcsb.org/pdb/home/home.do)

3) Sippl, M. J. Calculation of conformational ensembles from potentials of mean force. An approach to the knowledge-based prediction of local structures in globular proteins. *J Mol Biol* **213**, 859-883 (1990).

4) Sippl, M. J. Recognition of errors in three-dimensional structures of proteins. *Proteins* **17**, 355-362 (1993).

5) Laskowski, R. A., MacArthur, M. W., Moss, D. S. & Thornton, J. M. PROCHECK: a program to check the stereochemical quality of protein structures. *Journal of Applied Crystallography* **26**, 283-291, doi:doi:10.1107/S0021889892009944 (1993).

6) Xia, J., Sinelnikov, I., Han, B., and Wishart, D.S. (2015) MetaboAnalyst 3.0 – making metabolomics more meaningful . Nucl. Acids Res. 43, W251-257

7) Chatterjee, S., and A. S. Hadi. "Influential Observations, High Leverage Points, and Outliers in Linear Regression. *Statistical Science*. 1, 379–416 (1986).
